# Mitochondria-targeted phototherapeutic system enabling spatiotemporal-controlled NADH depletion for keloid intervention

**DOI:** 10.1016/j.mtbio.2026.103427

**Published:** 2026-06-30

**Authors:** Fugang Xiao, Xiang Cheng, Jianbo Chen, Yanpeng Fang, Xingru Zhou, Conghui Liu, Zhibing Fu, Ningling Wu, Yifei Xie, Lu Zhou, Shenming Xu, Wenbing Zeng, Jinrong Zeng

**Affiliations:** a Department of Dermatology, The Third Xiangya Hospital, Central South University, Changsha, 410013, PR China; b Xiangya School of Pharmaceutical Sciences, Central South University, Changsha, 410013, PR China

**Keywords:** Keloid, Photodynamic therapy, Platinum (II) complex, mtDNA damage, Apoptosis

## Abstract

Keloids represent a fibroproliferative disorder characterized by excessive collagen deposition and dysregulated cellular metabolism, yet effective therapeutic strategies remain limited due to the lack of precise intervention at the subcellular level. Herein, we report a mitochondria-targeted phototherapeutic system (**TBQQPt**) that enables spatiotemporal-controlled metabolic disruption through light-triggered NADH depletion. Unlike conventional photosensitizers that primarily rely on reactive oxygen species (ROS) generation, **TBQQPt** integrates mitochondrial localization with redox intervention, allowing localized consumption of NADH and amplification of oxidative stress within mitochondria. Mechanistically, **TBQQPt** accumulates in mitochondria and, upon light irradiation, induces efficient NADH oxidation, leading to mitochondrial dysfunction, enhanced ROS generation, and subsequent fibroblast inactivation. This dual mechanism establishes a self-amplifying therapeutic cascade that disrupts cellular energy metabolism and redox homeostasis. In vitro studies demonstrate significant inhibition of fibroblast proliferation and collagen production. In vivo, **TBQQPt**-mediated phototherapy effectively suppresses keloid progression with minimal off-target toxicity, as further supported by transcriptomic analysis revealing downregulation of fibrosis- and metabolism-related pathways. Overall, this work provides a subcellularly targeted and spatiotemporally controllable strategy for keloid treatment, highlighting the potential of integrating drug delivery with metabolic intervention for precision therapy of fibrotic diseases.

## Graphical abstract

A mitochondria-targeted phototherapeutic system (**TBQQPt**) enables spatiotemporally controlled activation upon light irradiation, inducing NADH depletion and amplifying reactive oxygen species (ROS) generation within mitochondria. This dual-action mechanism triggers mitochondrial dysfunction and metabolic collapse, leading to effective fibroblast inactivation and suppression of collagen deposition. The synergistic photodynamic–metabolic disruption ultimately results in significant keloid regression.

**Figure:**
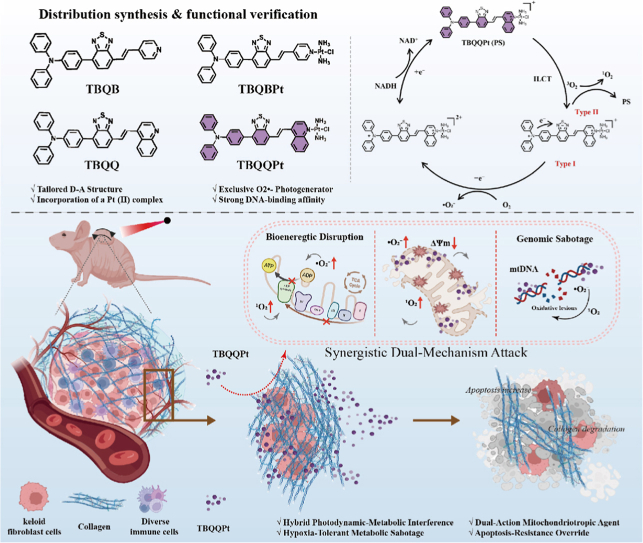

## 1 Introduction

Pathological fibroproliferative disorders, exemplified by keloids, represent a formidable clinical challenge characterized by aggressive peripheral expansion, high recurrence rates, and profound recalcitrance to conventional interventions [1,2]. Current therapeutic mainstays, ranging from surgical excision and intralesional corticosteroid injections to radiotherapy, are frequently undermined by suboptimal long-term efficacy, procedural invasiveness, and debilitating side effects [3]. This persistent therapeutic impasse necessitates the development of next-generation strategies capable of precisely dismantling the fundamental pathophysiological drivers that sustain keloid progression.

The resilience of keloids is increasingly attributed to a complex "*Warburg-like*" metabolic reprogramming within keloid fibroblasts (HKFs). To fuel their hyper-proliferative and hyper-synthetic state, HKFs undergo a metabolic shift toward aerobic glycolysis, a transition largely orchestrated by the PI3K/AKT signaling axis [4,5]. Despite this glycolytic reliance, mitochondrial integrity remains indispensable for HKF survival, providing the bioenergetic flexibility and redox buffering required to maintain the fibrotic phenotype [6,7]. This pathological network is further reinforced by elevated mitochondrial-derived reactive oxygen species (ROS), which function as persistent signaling molecules to drive extracellular matrix (ECM) deposition [8,9]. Recent multi-omics integration has identified key genetic hubs linking this oxidative stress to immune infiltration and apoptosis resistance, the latter often mediated by aberrant p53 phosphorylation [10]. Collectively, these interconnected mechanisms establish a self-sustaining pathological circuit that shields HKFs from standard pro-apoptotic stimuli.

This mechanistic landscape unveils a strategic vulnerability: disrupting the mitochondrial core could decisively collapse the HKF survival network. Photodynamic therapy (PDT) offers a compelling, spatially controlled approach to achieving this via the light-activated generation of cytotoxic ROS [11]. By overriding endogenous pathological signaling with a potent therapeutic oxidative burst, PDT can potentially bypass intrinsic apoptosis resistance. However, its translation to keloid therapy is hindered by two critical bottlenecks. First, the dense, fibrotic architecture creates a severely hypoxic microenvironment that compromises the efficacy of oxygen-dependent Type-II (singlet oxygen) pathways [12]. Second, conventional photosensitizers (PSs) predominantly rely on short-lived, diffusible ROS, which lack the sub-organelle precision required to effectively sabotage the specific bioenergetic machinery of the HKF [13].

To circumvent these limitations, although various targeted photosensitizers and nanomedicines have recently emerged to precision-treat fibrotic diseases by eliminating hyperactive fibroblasts or remodeling the extracellular matrix [14,15], most still heavily rely on oxygen-dependent pathways. Consequently, advanced photosensitizer engineering has pivoted toward oxygen-independent mechanisms and organelle-specific targeting. The design of Type-I PSs, which operate via electron transfer to generate superoxide radicals (•O_2_^−^), offers a viable route for sustained efficacy under low oxygen tension [16]. Furthermore, direct targeting of mitochondria, the powerhouse of the cell, promises enhanced specificity [17]. Platinum(II) and other heavy-metal complexes have emerged as ideal scaffolds for this purpose; they facilitate efficient intersystem crossing (ISC) via the heavy-atom effect and can be functionalized with lipophilic cationic motifs for specific mitochondrial accumulation [18,19]. However, the application of these sophisticated metal complexes has remained largely confined to oncology. A significant gap exists for a single-agent platform capable of a coordinated intramitochondrial assault that simultaneously paralyzes bioenergetic function and compromises genomic integrity within fibrotic tissues.

Inspired by this rationale, we designed a novel platinum (II)-coordinated donor-acceptor photosensitizer, **TBQQPt**. Its molecular architecture is precisely engineered to integrate mitochondrial tropism with a hybrid ROS generation profile and a unique capacity for photocatalytic electron abstraction. We hypothesized that upon photoactivation, **TBQQPt** would initiate a lethal intramitochondrial cascade within HKFs: initially hijacking the electron transport chain to induce an acute bioenergetic crisis, followed by direct oxidative damage to mitochondrial DNA (mtDNA), ultimately precipitating a "redox catastrophe" that triggers the intrinsic apoptotic pathway.

**Scheme 1:**
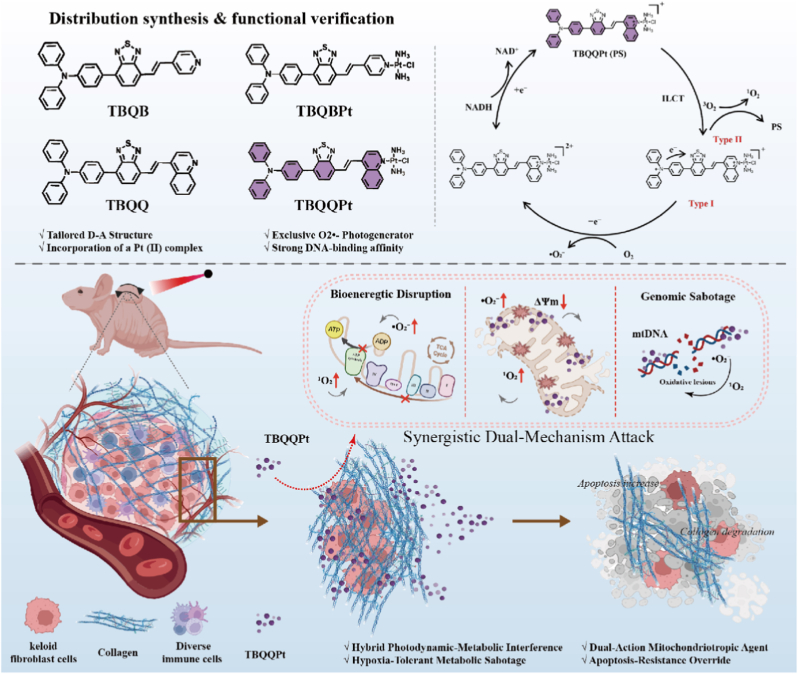
Schematic illustration of the stepwise preparation of **TBQQPt**, the proposed mechanism, and the therapeutic mechanism of **TBQQPt**-PDT against keloids.

This study provides a comprehensive validation of this hypothesis through a multidisciplinary framework. We characterize **TBQQPt**'s superior photophysical properties, demonstrate its sub-organelle localization, and confirm its potent in vitro cytotoxicity driven by mtDNA sabotage and metabolic failure. Distinct from conventional oncology-focused Pt-PSs that rely primarily on oxygen-consuming Type-II pathways, our design utilizes the Pt (II) center to drive a Type-I electron-transfer cascade. This system achieves therapeutic efficacy under hypoxia not just by generating radical species, but by actively abstracting electrons from NADH, thereby turning a conventional PDT agent into a metabolic disruptor tailored for the hypoxic, fibrotic microenvironment of keloids. Furthermore, the therapeutic efficacy of **TBQQPt** is rigorously evaluated in a patient-derived keloid xenograft (PDX) model, with genome-wide transcriptomic profiling elucidating the underlying molecular mechanisms. Ultimately, this work establishes **TBQQPt** as a promising therapeutic candidate and introduces a novel paradigm of "metabolic interference coupled with genomic sabotage" for the precision treatment of metabolically aberrant fibroproliferative diseases (Scheme 1).

## 2 Results and discussion

### 2.1 Design, synthesis, and fundamental photophysical properties of TBQQPt

**Fig. 1:**
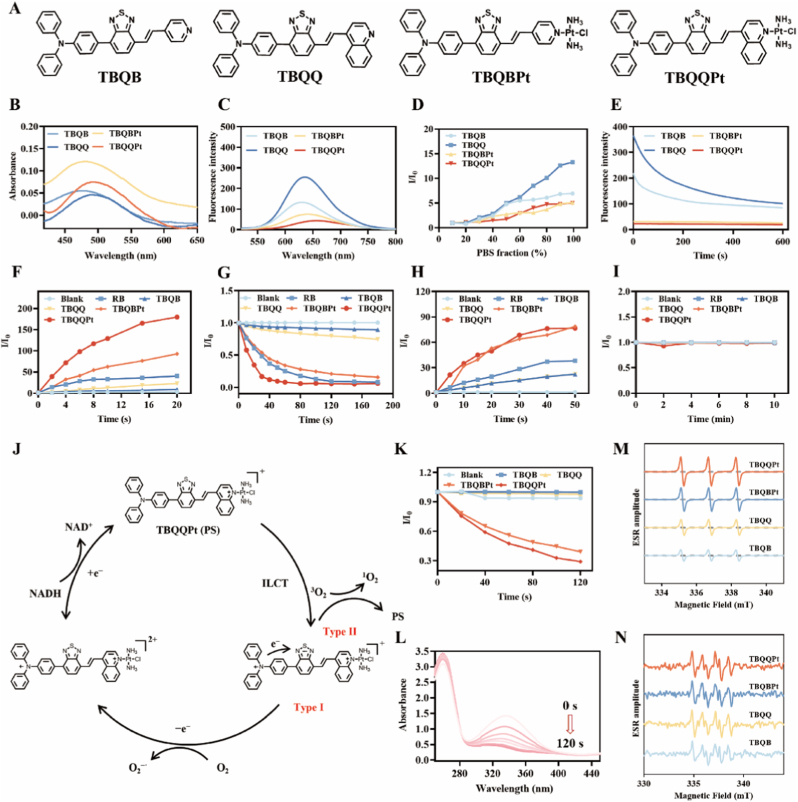
**Photophysical properties of TBQB, TBQQ, TBQBPt, and TBQQPt. A)** Chemical structures of **TBQB**, **TBQQ**, **TBQBPt**, and **TBQQPt**. **B)** UV–vis absorption spectra of **TBQB**, **TBQQ**, **TBQBPt**, and **TBQQPt** (10 μM) recorded in PBS solution. **C)** Fluorescence emission spectra of **TBQB**, **TBQQ**, **TBQBPt**, and **TBQQPt** (10 μM) in PBS solution. **D)** Relative fluorescence intensity (I/I_0_) of **TBQB**, **TBQQ**, **TBQBPt**, and **TBQQPt** in DMSO/PBS mixtures with different PBS volume fractions, where I and I_0_ represent the fluorescence intensities in DMSO/PBS mixtures and pure DMSO, respectively. **E)** Photostability of **TBQB**, **TBQQ**, **TBQBPt**, and **TBQQPt** under continuous light irradiation. **F)** Relative fluorescence intensity (I/I_0_) of DCFH at 530 nm under white light irradiation (20 mW cm^−2^) for different treatment groups. **G)** Relative absorbance change (A – A^0^) of ABDA at 408 nm under white light irradiation (20 mW cm^−2^) for different treatment groups. **H)** Relative fluorescence intensity (I/I_0_) of DHR123 at 535 nm under white light irradiation (20 mW cm^−2^) for different treatment groups. **I)** Relative fluorescence intensity (I/I_0_) of 3-CCA at 418 nm under white light irradiation (20 mW cm^−2^) for different treatment groups. **J)** Schematic illustration of the possible mechanisms for simultaneous ROS generationand NADH oxidation catalyzed by **TBQQPt**. ILCT: intraligand charge transfer. **K)** Relative absorbance change (A/A_0_) of NADH at 340 nm under white light irradiation (20 mW cm^−2^) for different treatment groups. **L)** UV–vis absorption spectra of NADH co-incubated with **TBQQPt** (10 μM) under white-light irradiation. **M)** ESR spectra of TEMP/^1^O_2_ adducts under white light irradiation (20 mW cm^−2^). **N)** ESR spectra of DMPO/O_2_^•−^ adducts under white light irradiation (20 mW cm^−2^).

The hypoxic and fibrotic microenvironment of keloids, coupled with the apoptosis resistance of hyperactive fibroblasts, presents a significant therapeutic challenge [20]. While PDT offers a localized treatment modality, its clinical efficacy is often limited by the reliance of conventional PSs on oxygen-dependent Type-II reactions and a lack of subcellular specificity [21]. To overcome these barriers, our design was rooted in donor-acceptor (D-A) structured organic scaffolds, widely recognized for their favorable light-harvesting and tunable electronic properties [22]. We initially synthesized two D-A organic PSs, **TBQB** and **TBQQ (**Fig. 1A**)**, featuring defined donor-acceptor separation to facilitate intramolecular charge transfer (ICT) upon photoexcitation, endowing the excited states with intrinsic electron-accepting characteristics advantageous for photoinduced electron-transfer processes. However, purely organic PSs often encounter efficiency bottlenecks in electron transfer and limited interactions with biological macromolecules, restricting their ability to disrupt the robust metabolic pathways of keloid fibroblasts [23].

To enhance metabolic perturbation, we employed a transition-metal strategy. While preserving the conjugated backbone, Pt (II) centers were incorporated to construct the metallated derivatives **TBQBPt** and **TBQQPt** (Fig. 1A; Scheme S1). Pt (II) possesses strong electronic regulatory capability; its coordination effectively reconstructs the frontier molecular orbital distribution of the PS. This modification enhances the electron-accepting character of the photoexcited state, facilitating more efficient participation in electron-transfer processes, such as competitively extracting electrons from metabolic cofactors like NADH [24]. This transition transforms the PSs from a generator of nonspecific oxidative damage into a metabolically disruptive agent capable of targeting mitochondrial respiration at the electron-transfer level. All compounds were obtained in high purity and fully characterized (Figs. S1–S12).

The photophysical properties of series (10 μM in PBS) were systematically evaluated. The UV-Vis absorption spectra showed broad absorption bands from 400 to 600 nm for all compounds (Fig. 1B), suitable for visible light activation. Solvent-dependent studies (Figs. S13–S14) confirmed pronounced ICT behavior. A key observation was that Pt (II) incorporation induced a distinct red-shift in fluorescence emission maxima, by approximately 30 nm, with **TBQQPt** emitting at 683 nm (Fig. 1C). This is attributed to Pt-induced stabilization of the ICT excited state, enhancing its electron-accepting character [25]. Owing to the triphenylamine donor, all compounds exhibited pronounced aggregation-induced emission (AIE) characteristics, with fluorescence intensity surging in DMSO/PBS mixtures as the PBS fraction increased (Fig. 1D; Fig. S15). This ensures high emissivity in the aggregated cellular state. Furthermore, **TBQQPt** demonstrated superior photostability under continuous irradiation compared to its organic analogues (Fig. 1E; Fig. S16), a vital requirement for sustained therapeutic effect.

We next evaluated the core photodynamic function. Using DCFH-DA as a general ROS probe under white light irradiation (20 mW cm^−2^), the Pt-coordinated PSs exhibited significantly enhanced total ROS generation compared to their organic counterparts. **TBQQPt** displayed the strongest capability, outperforming the commercial PS Rose Bengal (RB) (Fig. 1F; Fig. S17). This enhancement is attributed to the stronger electron-withdrawing quinoline unit in **TBQQPt**, which reinforces donor-acceptor asymmetry and facilitates charge redistribution, an effect potentiated by Pt coordination. To further elucidate the ROS generation efficiency of **TBQQPt** and its intrinsic photophysical mechanism at the molecular level, systematic quantum chemical calculations were performed to investigate the excited-state processes. **TBQQPt** (10 μM) efficiently catalyzed the degradation of ABDA (50 μM), confirming robust singlet oxygen (^1^O_2_) production via a Type-II mechanism, with a rate more than twice that of RB (Fig. 1G; Fig. S18). Concurrently, it generated substantial superoxide anion (•O_2_^−^), as evidenced by the oxidation of DHR123 (10 μM), significantly outperforming both RB and the organic analogues (Fig. 1H; Fig. S19). This unequivocally established the Pt-coordinated PSs, especially **TBQQPt**, as enhanced hybrid Type-I/II photosensitizers, with Pt incorporation playing a decisive role in activating efficient electron-transfer-driven (Type-I) pathways. Hydroxyl radical (•OH) generation was not detected under these conditions (Fig. S20). The generation of both ^1^O_2_ and •O_2_^−^ was corroborated by characteristic TEMP-^1^O_2_ and DMPO-•O_2_^−^ ESR signals (Fig. 1M and N). This dual-pathway capability ensures potent oxidative damage across tissue oxygen gradients.

A distinctive and mechanistically pivotal feature of this system was the Pt-enabled photocatalytic oxidation of NADH. In a DMF/PBS system (to avoid solvent interference), the organic PSs **TBQB** and **TBQQ** failed to induce appreciable NADH (200 μM) photooxidation. In sharp contrast, both **TBQBPt** and **TBQQPt** (10 μM) under white light irradiation (20 mW cm^−2^) demonstrated pronounced activity, with the characteristic NADH absorption at 340 nm decreasing rapidly (Fig. 1K and L; Fig. S21). This underscores the decisive role of Pt center in elevating electron-transfer efficiency to a lever that can actively compete with biological redox systems.

We propose a synergistic reaction model (Fig. 1J): Photoexcitation of **TBQQPt** generates an ICT state. Owing to the presence of the Pt (II) center, intersystem crossing is effectively promoted via the heavy-atom effect, leading to the formation of a long-lived excited photosensitizer state (PS). In the Type-I pathway, PS can undergo photoinduced electron transfer to O_2_, producing •O_2_^−^. Concurrently, the oxidized PS species (**TBQQPt**•^+^) can be regenerated by abstracting an electron from NADH, oxidizing it to NAD^+^. In the parallel Type-II pathway, PS transfers energy to O_2_, yielding ^1^O_2_. This dual-pathway mechanism enables **TBQQPt** to simultaneously generate ROS and deplete the key redox cofactor NADH, imposing a comprehensive redox catastrophe.

### 2.2 Photochemical mechanism and electronic structure basis of TBQQPt

**Fig. 2:**
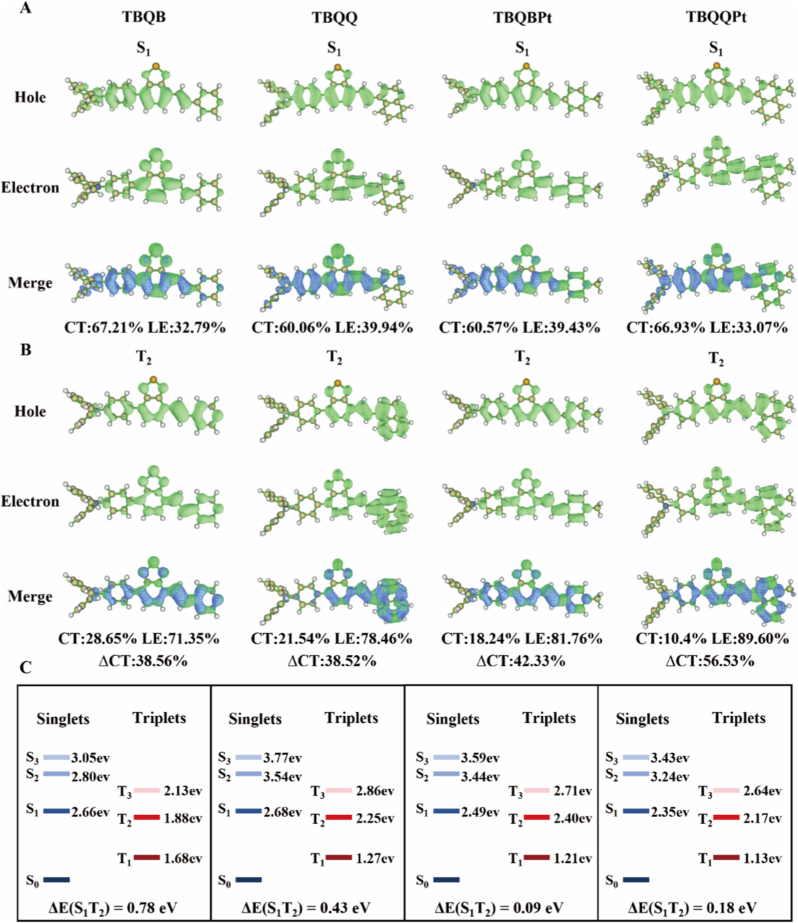
**Electron-hole analysis of the S_1_and T_1_states of TBQB, TBQQ, TBQBPt and TBQQPt. A)** Hole, electron and electron-hole diagram of **TBQB, TBQQ, TBQBPt** and **TBQQPt** in S_1_ state. **B)** Hole, electron and electron-hole diagram of **TBQB, TBQQ, TBQBPt** and **TBQQPt** in T_2_ state. **C)** Energy level difference of a single excited state and triple excited state.

To elucidate the electronic-structure origins of the enhanced performance, DFT/TD-DFT calculations were performed. Geometrical optimization revealed pronounced torsional distortion in all molecules, consistent with AIE characteristics and beneficial for suppressing non-radiative decay. Analysis of the electron-hole distributions for the S_1_ and T_2_ excited states revealed that all compounds exhibit mixed charge-transfer (CT) and locally excited (LE) characteristics (Fig. 2A and B). Quantitatively, upon transition from S_1_ to T_2_, the CT contribution decreases while the LE contribution increases (e.g., ΔCT% = −42.33% for **TBQBPt**, −56.53% for **TBQQPt**). This shift toward LE character in the triplet state favors efficient intersystem crossing (ISC) according to El-Sayed's rule [26]. Crucially, the calculated energy gap between the lowest singlet (S_1_) and triplet (T_1_) states (ΔE_ST_) was smallest for **TBQQPt** (Fig. 2C). Furthermore, the S_1_–T_2_ energy gaps were relatively small for all compounds (e.g., 0.18 eV for **TBQQPt**). Small singlet-triplet gaps are fundamental for enhancing ISC efficiency, providing a coherent rationale for the superior ROS generation of the Pt-coordinated PSs, particularly **TBQQPt**.

Collectively, the structure-activity relationship (SAR) of Pt (II) coordination reveals a synergistic dual effect. Physically, the Pt (II) heavy-atom effect reduces the ΔE_ST_ to accelerate ISC, directly amplifying ROS generation. Electronically, its strong electron-withdrawing nature stabilizes the ICT state. This stabilization not only red-shifts the emission but also provides the thermodynamic driving force required to abstract electrons from NADH. Thus, Pt (II) coordination fundamentally upgrades the organic scaffold from a conventional ROS generator into an active metabolic disruptor.

**Fig. 3:**
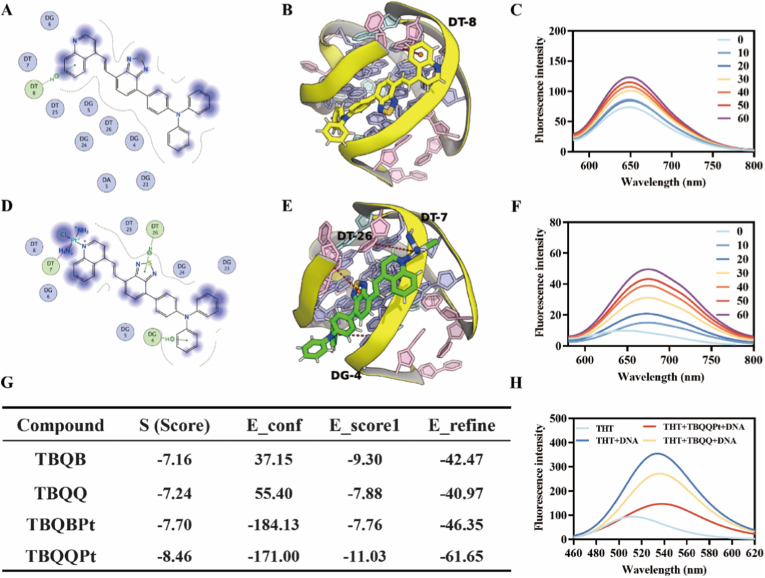
**A)** Molecular docking of **TBQQ** (2D). **B)** Molecular docking of **TBQQ**(3D). **C)** Emission spectra of **TBQQ** (10 μM) titrated with dsDNA in Tris-HCl buffer. **D)** Molecular docking of **TBQQPt** (2D). **E)** Molecular docking of **TBQQPt** (3D). **F)** Emission spectra of **TBQQPt** (10 μM) titrated with dsDNA in Tris-HCl buffer. **G)** Docking score results of **TBQB**, **TBQQ**, **TBQBPt**, and **TBQQPt** interacting with DNA. *S* (Score) represents the final docking score. *E*(conf) denotes the conformer energy of the ligand. *E*(score1) corresponds to the binding score calculated at the initial placement stage prior to structural optimization. *E*(refine) represents the interaction energy calculated during the refinement stage of docking. **H)** Fluorescence spectra of THT after different treatments.

Given the planar aromatic structure and the Pt (II) center's known DNA affinity [27], we investigated interactions with dsDNA. Molecular docking predicted that **TBQQPt**, through its Pt center, forms a tighter, more integrated interaction with the DNA helix (e.g., via DT7, DT26, DG4 sites) compared to the looser binding of **TBQQ** (Fig. 3A, B, D, E). This was quantified by lower (i.e., better) docking scores for the Pt complexes (Fig. 3G). Experimentally, fluorescence titration (10 μM PS in Tris-HCl buffer) showed that **TBQQPt** exhibited a ∼5.9-fold emission enhancement upon dsDNA addition, significantly greater than the ∼1-fold increase for **TBQQ** (Fig. 3C–F), confirming stronger DNA interaction. A competitive assay using thioflavin T (THT) further showed that **TBQQPt** effectively displaced THT from DNA, while **TBQQ** had a minimal effect (Fig. 3H). These results consistently demonstrate that Pt chelation markedly strengthens the DNA-binding ability of the PS.

### 2.3 Cellular uptake, mitochondrial specificity, and induction of apoptosis

**Fig. 4:**
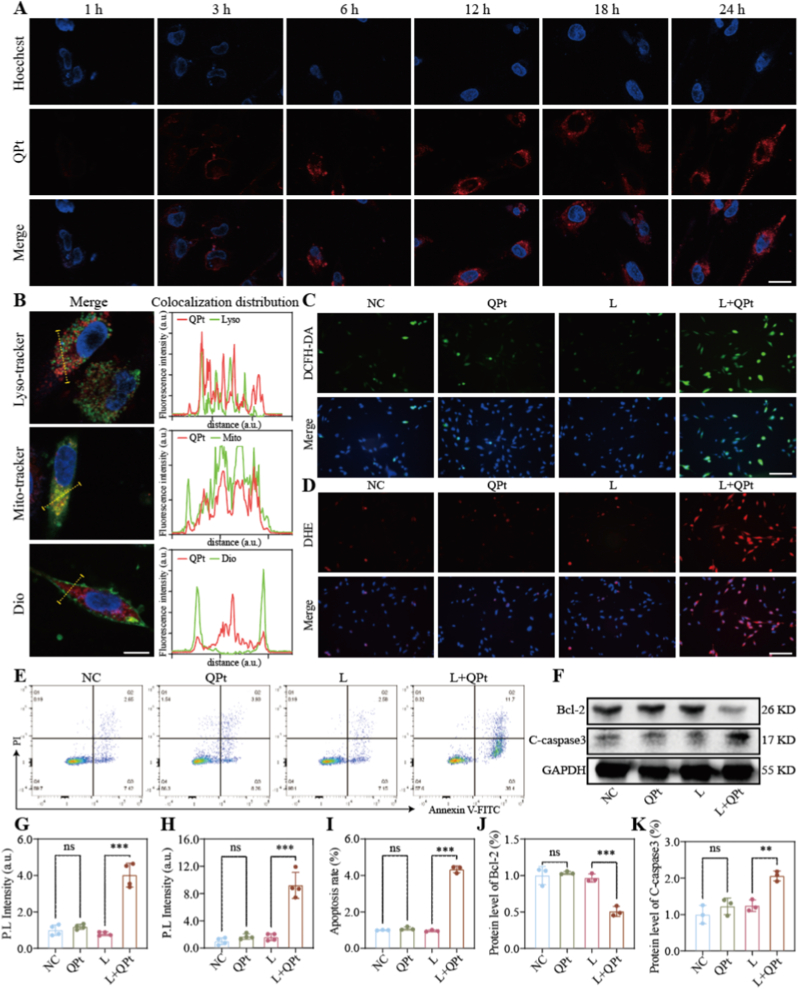
**Uptake characteristics and cellular functions of TBQQPt *in vitro*. A)** Cellular uptake CLSM images of HKFs stained by Hoechst 33342 and **TBQQPt** (8 μM). Scale bar: 20 μm. **B)** Colocalization images of HKFs co-stained with **TBQQPt** (8 μM) and different commercial dyes, including Lyso-tracker Green (0.1 μM), Mito-tracker Green (1 μM), DiO (1 μM), and Hoechst 33342 (1 μM). Scale bar: 10 μm. **C)** Detection of total cellular ROS using DCFH-DA. NC: control (PBS); **QPt**: **TBQQPt** without irradiation; L: irradiation alone; L + **QPt**: **TBQQPt** added followed by white light irradiation (White light, 20 mW cm^−2^, 5 min). Scale bar: 100 μm. **D)** DHE was used to detect •O_2_^−^ (White light, 20 mW cm^−2^, 5 min). Scale bar: 100 μm. **E)** Flow cytometry analysis of apoptosis in HKFs after different treatments, with Annexin V-FITC fluorescence intensity on the x-axis and PI fluorescence intensity on the y-axis (White light, 20 mW cm^−2^, 5 min). **F)** Representative Western blot images showing the protein levels of Cleaved caspase-3 (C-caspase3) and Bcl-2 in HKFs after the indicated treatments (White light, 20 mW cm^−2^, 5 min). **G)** Quantitative analysis of relative DCFH fluorescence intensity in cells, n = 4. **H)** Quantitative analysis of relative DHE fluorescence intensity in cells, n = 4. **I)** Statistical analysis of the total apoptosis rate, n = 3. **J)** Quantitative analysis of the protein of level Bcl-2 by western blot, n = 3. **K)** Quantitative analysis of the protein level of C-Caspase 3 by western blot, n = 3. The results are expressed as mean ± SD. ∗*p* < 0.05, *p* < 0.01, ∗*p* < 0.001; ns, not significant. (For interpretation of the references to colour in this figure legend, the reader is referred to the Web version of this article.)

To translate the superior photochemical properties of **TBQQPt** into a biological context, we first characterized its interaction with HKFs. Initial cell viability assays established a non-toxic working concentration for **TBQQPt** under dark conditions (Fig. S22A). Subsequently, the role of Pt (II) coordination in boosting phototherapeutic efficacy was verified by comparing the ligand **TBQQ** and the complex **TBQQPt** in HKFs. **TBQQ** exhibited excellent dark biocompatibility, with 64 μM determined as the non-toxic threshold (Fig. S26A). Under identical white light irradiation (20 mW cm^−2^, 5 min), **TBQQ** (64 μM) induced no detectable phototoxicity (Fig. S26B). Conversely, an 8-fold lower concentration of **TBQQPt** (8 μM) caused a dramatic drop in cell viability. This contrast confirms that Pt (II) coordination significantly elevates phototherapeutic potency, substantiating the rationality of our molecular design. We then investigated **TBQQPt**'s cellular internalization and subcellular fate. Confocal laser scanning microscopy (CLSM) confirmed the efficient and time-dependent uptake of **TBQQPt** at this concentration (Fig. 4A; Fig. S22B). To precisely determine its intracellular localization, systematic co-localization studies were conducted using organelle-specific fluorophores. The results revealed a striking overlap between the fluorescence signal of **TBQQPt** and MitoTracker Green, while revealing minimal association with markers for lysosomes (Lyso-tracker Green), the endoplasmic reticulum (DiO), or the nucleus (Hoechst 33342) (Fig. 4B). These data conclusively establish the predominant mitochondrial localization of **TBQQPt**. The targeting mechanism is attributed to its lipophilic cationic character, imparted by the Pt (II) center, which facilitates electrophoretic accumulation into the mitochondrial matrix driven by the negative inner membrane potential (ΔΨm) [28,29].

Notably, the hypoxic microenvironment commonly found in keloid tissues poses a significant challenge to traditional Type-II photodynamic therapy, which relies on oxygen for ^1^O_2_ generation [12,30]. To evaluate the potential of **TBQQPt**-PDT under such conditions, we first measured ROS generation. Using the broad-spectrum probe DCFH-DA, we found that **TBQQPt** combined with white light irradiation (20 mW cm^−2^, 5 min) triggered a pronounced burst of total intracellular ROS in HKFs (Fig. 4C–G). Further analysis with the •O_2_^−^-specific probe dihydroethidium confirmed the substantial generation of this species (Fig. 4D–H). The significant accumulation of •O_2_^−^ is a hallmark of Type-I photodynamic reactions, which proceed via an electron transfer pathway with a relatively lower dependence on molecular oxygen [31]. Therefore, **TBQQPt** demonstrates a hybrid Type-I/II ROS generation capability. This mixed mechanism is a strategic advantage, ensuring potent oxidative damage can be sustained even within the variable and often hypoxic oxygen tensions of pathological tissues. Furthermore, flow cytometric analysis using Annexin V-FITC and propidium iodide (PI) staining confirmed that the combination of **TBQQPt** and light (L + **QPt**) significantly induced apoptosis in HKFs (Fig. 4E–I). Western blot analysis revealed that the treatment led to a marked downregulation of the anti-apoptotic protein Bcl-2 and a concomitant cleavage/activation of the executioner caspase-3 (Fig. 4F–J, K). Similarly, cell viability analysis (using calcein-AM/PI) and cell migration ability detection under the same conditions further supported the above conclusion (Fig. S22C–F). Collectively, these results delineate a clear causal chain: precise mitochondrial targeting enables the in-situ generation of a hybrid ROS storm, which directly triggers the intrinsic apoptotic cascade to eliminate therapy-resistant HKFs.

### 2.4 Mechanisms of mitochondrial dysfunction

**Fig. 5:**
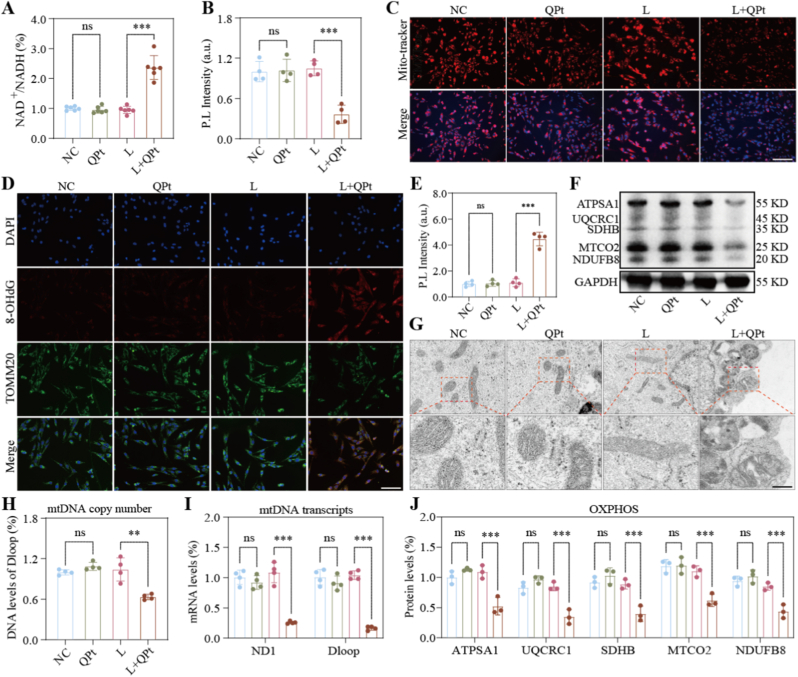
**Assessing the effects of TBQQPt on mitochondrial function and its mechanism of action *in vitro*. A)** The NAD+/NADH kit was used to conduct quantitative analysis of the destruction of the intracellular nicotinamide adenine dinucleotide pool caused by light induction (White light, 20 mW cm^−2^, 5 min), n = 6. **B)** Quantitative analysis of relative Mito-tracker Red CMXRos fluorescence intensity in cells, n = 4. **C)** Mito-tracker Red CMXRos was used to detect mitochondrial function (White light, 20 mW cm^−2^, 5 min). Scale bar: 100 μm. **D)** Representative fluorescence micrographs of HKFs co-stained with DAPI (blue, nuclei), anti-8-OHdG (red, mitochondrial DNA damage marker), and anti-TOMM20 (green, mitochondrial outer membrane) under different treatments (White light, 20 mW cm^−2^, 5 min). Scale bar: 100 μm. **E)** Quantitative analysis of relative 8-OHdG fluorescence intensity in cells, n = 4. **F)** The relative levels of mitochondrial OXPHOS in HKFs after intervention were measured by western blot (White light, 20 mW cm^−2^, 5 min). **G)** TEM images of HKFs after different treatments (White light, 20 mW cm^−2^, 5 min). Scale bar: 200 nm. **H)** mtDNA copy number in HKFs analyzed by RT-qPCR (White light, 20 mW cm^−2^, 5 min), n = 4. **I)** mtDNA transcript levels. mtDNA levels were normalized to the internal control actin. Mitochondrial genes ND1 and Dloop were chosen to indicate mtDNA transcription (White light, 20 mW cm^−2^, 5 min), n = 4. **J)** Quantitative analysis of protein levels of ATPSA1, UQCRC1, SDHB, MTCO2, and NDUFB8 by western blot, n = 3. The results are expressed as mean ± SD. ∗*p* < 0.05, *p* < 0.01, ∗*p* < 0.001; ns, not significant. (For interpretation of the references to colour in this figure legend, the reader is referred to the Web version of this article.)

We next sought to elucidate the critical upstream events that commit HKF mitochondria to apoptosis following **TBQQPt**-PDT. Initially, to verify whether the photocatalytic NADH oxidation observed in solution effectively translates into cellulo metabolic disruption, we directly quantified the intracellular concentrations of NADH and NAD^+^ in HKFs following various treatments. We confirmed that the combination of **TBQQPt** and light irradiation induced a remarkable, steep depletion of intracellular NADH, accompanied by a synchronous elevation in NAD^+^ levels (Fig. S24B and C). This metabolic shift resulted in a multi-fold surge in the intracellular NAD^+^/NADH ratio (Fig. 5A). This direct cell-based evidence explicitly confirms that TBQQPt acts as an efficient intramitochondrial photocatalyst, successfully redirecting electronic flux to clear cellular NADH currency and establishing a severe bioenergetic crisis. Subsequently, further assessment of mitochondrial integrity revealed a rapid and profound dissipation of the mitochondrial membrane potential (ΔΨm), as indicated by diminished MitoTracker Red CMXRos fluorescence, which serves as a key indicator of functional health (Fig. 5B and C). This loss of ΔΨm was corroborated ultrastructurally by TEM, which visualized severely swollen mitochondria with disintegrated cristae, a morphological hallmark of irreversible damage (Fig. 5G). We hypothesized that the unique properties of **TBQQPt**, mitochondrial accumulation, potent ROS generation, and inherent DNA-binding affinity, would converge to inflict precise damage upon mtDNA. This hypothesis was confirmed by immunofluorescence co-staining, which demonstrated a massive and mitochondria-specific increase in 8-hydroxy-2′-deoxyguanosine, an established marker of oxidative DNA damage, with strong spatioal co-localization to the mitochondrial matrix as marked by TOMM20 (Fig. 5D and E).

This targeted assault on mtDNA constitutes a mechanistic cornerstone of **TBQQPt**-PDT, distinguishing it from conventional photosensitizers that mediate nonspecific cytotoxicity via diffusible ROS. By generating high concentrations of ROS in immediate proximity to mtDNA, **TBQQPt** effects a site-specific and potent genomic insult. To investigate the therapeutic mechanism, the light-induced DNA cleavage capability of **TBQQPt** was evaluated via agarose gel electrophoresis using pUC-19 plasmid DNA. Under white light irradiation, **TBQQPt** induced concentration-dependent DNA cleavage (Fig. S24A). While the plasmid remained intact at low concentrations, the major dsDNA bands at 2686 bp progressively degraded as the **TBQQPt** concentration increased from 5 to 50 μM, disappearing completely at the highest concentration. These results confirm that efficient photodynamic DNA cleavage contributes significantly to the potent phototoxicity of **TBQQPt**. Given the documented metabolic dysregulation and apoptotic resistance of keloid fibroblasts, this strategy of directly compromising the genetic core of their bioenergetic apparatus represents a particularly effective therapeutic approach [32,33]. The functional consequences of this mtDNA damage were severe and multifaceted. Quantitative PCR analysis confirmed a significant reduction in both mtDNA copy number and the transcription of key mtDNA-encoded genes, including ND1 (a component of Complex I) and the regulatory D-loop region (Fig. 5H and I). As mtDNA encodes essential subunits of the oxidative phosphorylation (OXPHOS) system, this transcriptional collapse precipitated a wholesale failure of cellular respiration. Correspondingly, western blot analysis revealed a coordinated downregulation of representative protein subunits across all five OXPHOS complexes (I–V) (Fig. 5F–J). This pattern is consistent with a failure in de novo protein synthesis arising from the primary genomic damage, rather than from nonspecific proteolytic degradation. The resulting bioenergetic crisis, characterized by a collapse in ATP production coupled with exacerbated ROS emission, establishes a lethal and self-amplifying cycle that irrevocably commits the HKFs to apoptotic death [34,35].

### 2.5 Therapeutic efficacy in a patient-derived keloid xenograft model in vivo

**Fig. 6:**
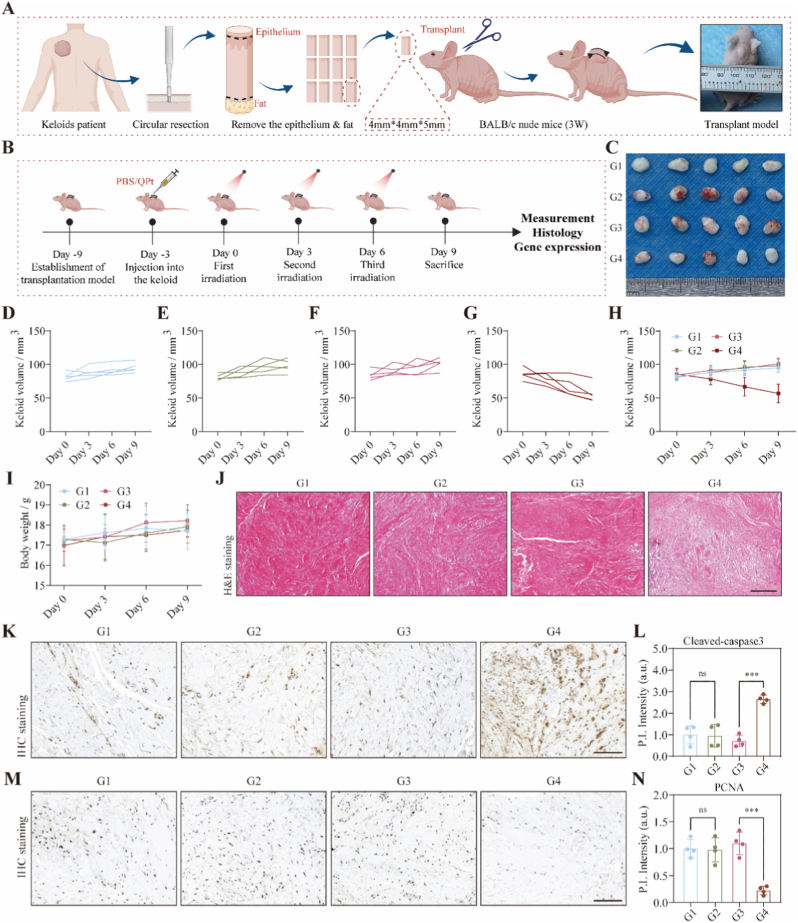
**Assessing the efficacy of TBQQPt-mediated photodynamic therapy in keloid xenograft mouse model. A)** Flowchart of the patient-derived xenograft keloid mouse model establishment. **B)** Schematic diagram of the in vivo PDT protocol mediated by **TBQQPt** (200 μM, white light irradiation (White light, 20 mW cm^−2^ for 10 min). **C)** Representative photographs of keloids from each treatment group on day 9. G1: Control (PBS); G2: **TBQQPt** injection without irradiation; G3: Irradiation alone; G4: **TBQQPt** injection followed by irradiation. **D-G)** Comparative analysis of individual keloid growth kinetics in mice under different treatments, n = 5. **H)** Quantification of average keloid volume across treatment groups, n = 5. **I)** Body weight curves for all groups following various treatments. **J)** Representative images of H&E-stained keloid tissue sections from different treatment groups. Scale bar: 200 μm. **K)** Representative immunohistochemical staining for cleaved-Caspase 3 in keloid tissues across treatment groups. Scale bar: 200 μm. **L)** Quantitative analysis of cleaved Caspase-3 immunoreactivity in keloid tissue was performed, n = 4. **M)** Representative immunohistochemical staining for PCNA in keloid tissues across treatment groups. Scale bar: 200 μm. **N)** Quantitative analysis of PCNA immunoreactivity in keloid tissue was performed, n = 4. The results are expressed as mean ± SD. ∗*p* < 0.05, *p* < 0.01, ∗*p* < 0.001; ns, not significant.

The therapeutic potential of this coherent cellular mechanism was evaluated in a clinically stringent, patient-derived keloid xenograft mouse model [36]. This model is particularly stringent as it preserves the native human tissue architecture and the complex stromal interactions characteristic of the disease (Fig. 6A). To further investigate the in vivo behavior of **TBQQPt** after local administration, fluorescence imaging was performed following subcutaneous injection. As shown in Fig. S27, a distinct fluorescence signal was observed at the injection site immediately after administration and remained detectable for at least 24 h, although with a gradual decrease in intensity over time. The prolonged local retention of **TBQQPt** is beneficial for sustained photodynamic action and minimizes rapid systemic diffusion, thereby favoring localized therapeutic efficacy while reducing potential off-target exposure. These findings support the suitability of **TBQQPt** for local phototherapy applications in keloid treatment.

Following a standardized protocol, intralesional injection of **TBQQPt** (group G4) followed by localized white light irradiation (20 mW cm^−2^ for 10 min) resulted in significant suppression of keloid growth over a 9-day period (Fig. 6B and C). The synergistic L + **QPt** treatment group markedly outperformed all control cohorts, including the PBS control (G1), drug-only (G2), and light-only (G3) groups (Fig. 6D–H). Crucially, the regimen was well-tolerated, with no adverse effects on mouse body weight observed in any group (Fig. 6I) and no evidence of systemic toxicity upon histopathological examination of major organs (Heart, liver, spleen, lung, kidney, brain) (Fig. S23). Additionally, the biocompatibility of **TBQQPt** was evaluated via in vitro hemolysis assay (5–400 μM). The hemolysis ratio remained well below the 5% safety threshold, indicating negligible red blood cell damage (Fig. S25A). In vivo serum analysis from treated mice showed no significant differences in liver (ALT, AST) and kidney (Urea, Cr) markers, nor in the inflammatory cytokine TNF-α, compared to healthy controls (Fig. S25B–F). These results confirm that **TBQQPt** is safe for systemic use, causing no organ dysfunction or severe immune response. This profile highlights the high safety margin and spatial precision of the PDT approach.

Comprehensive analysis of the excised keloid tissues provided compelling in vivo validation of the proposed mechanism. In Group G4, H&E staining revealed a noticeable reduction in collagen density and organization compared to the other groups (Fig. 6J). Similarly, immunohistochemical staining confirmed a significant increase in the number of cleaved-caspase 3 positive cells within the keloid specimens of Group G4 (Fig. 6K and L), providing direct in situ evidence for light-triggered apoptosis. Furthermore, staining for the proliferation marker PCNA was markedly reduced in the G4 group (Fig. 6M and N), indicating that **TBQQPt**-PDT not only kills existing keloid fibroblasts but also suppresses the proliferative drive of the residual population. By targeting both the survival and the reproduction of these cells, **TBQQPt**-PDT addresses the fundamental drivers of keloid persistence and recurrence.

### 2.6 Transcriptomic profiling reveals a coordinated suppression of metabolism and activation of apoptosis

**Fig. 7:**
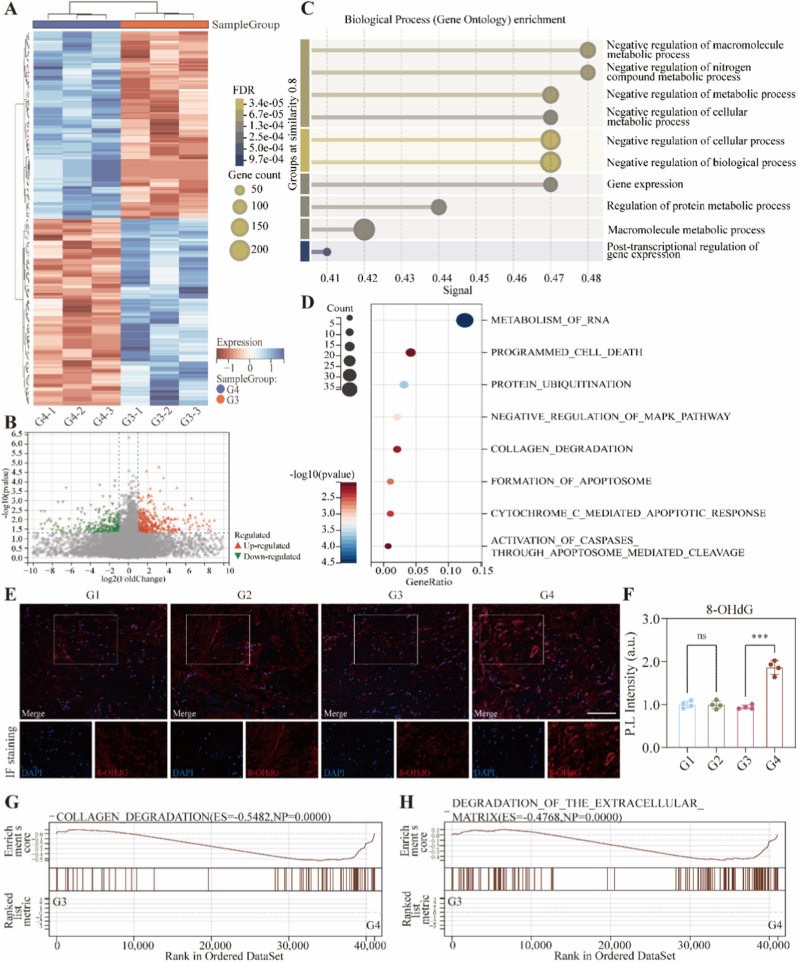
**Transcriptomics unravels the mechanism of TBQQPt-PDT in a keloid xenograft model. A)** Heat map of DEGs (adj. p-value <0.05 and based on RNA-seq results). **B)** Volcano plot of DGEs (|log_2_ fold change| ≥ 1). **C)** GO enrichment analysis (Biological Process) of DGEs using the STING database. **D)** KEGG pathway enrichment of DEGs. **E)** Representative immunofluorescence images of xenograft keloid tissue. Sections were co-stained with DAPI (blue, nuclei) and an antibody against 8-OHdG (red, a marker of mitochondrial DNA damage) under the indicated treatment conditions. Scale bar: 100 μm. **F)** Quantitative analysis of relative 8-OHdG fluorescence intensity in tissues, n = 4. **G, H)** Gene Set Enrichment Analysis was performed on RNA-seq data to compare the G3 and G4 groups (adj. p-value <0.01). The results are expressed as mean ± SD. ∗*p* < 0.05, *p* < 0.01, ∗*p* < 0.001; ns, not significant. (For interpretation of the references to colour in this figure legend, the reader is referred to the Web version of this article.)

To elucidate the genome-wide transcriptional alterations induced by **TBQQPt**-PDT in vivo, we performed RNA-seq on harvested patient-derived xenograft tissues. Principal component analysis revealed a distinct separation between the transcriptomic profile of the treatment group (G4) and the light-only control group (G3), demonstrating that the therapy instigates a specific and profound transcriptional reprogramming. Differential expression analysis (adjusted p-value <0.05, |log_2_ fold change| ≥ 1) further identified a substantial set of significantly dysregulated genes in the G4 group (Fig. 7A and B).

To decipher the biological implications of these changes, we performed Gene Ontology (GO) enrichment analysis on the differentially expressed genes. Notably, the most significantly enriched terms in the G4 group were overwhelmingly related to the global negative regulation of cellular functions. This included broad processes such as "negative regulation of biological process," "negative regulation of cellular process," and "negative regulation of metabolic process," as well as more specific terms like "negative regulation of macromolecule metabolic process" and "negative regulation of nitrogen compound metabolic process" (Fig. 7C). Concurrently, terms related to core cellular activities such as "gene expression," "regulation of protein metabolic process," "macromolecule metabolic process," and "post-transcriptional regulation of gene expression" were also enriched. This collective signature strongly suggests that **TBQQPt**-PDT exerts its effect primarily by inducing a broad-scale suppression of cellular metabolism and associated biological processes, consistent with a state of bioenergetic and biosynthetic shutdown.

To identify specific activated or inhibited pathways driving cell death, we conducted Kyoto Encyclopedia of Genes and Genomes (KEGG) pathway analysis. This revealed a highly significant enrichment of pathways directly associated with intrinsic (mitochondrial) apoptosis, including "cytochrome c-mediated apoptotic response" and "apoptosome assembly" (Fig. 7D). This systems-level evidence independently validates our core conclusion that **TBQQPt**-PDT primarily induces cell death via the mitochondrial pathway. The persistence of this central mechanism in vivo was further confirmed by elevated levels of the mtDNA oxidative damage marker 8-OHdG within the treated keloid tissues (Fig. 7E and F).

Intriguingly, Gene Set Enrichment Analysis (GSEA) also indicated a significant shift in gene sets related to the "degradation of the extracellular matrix organization" (Fig. 7G and H). While the precise cellular origin (e.g., residual fibroblasts versus infiltrating immune cells) and functional consequences of these transcriptomic changes require further mechanistic investigation, this intriguing finding hints at a secondary effect beyond direct fibroblast killing. It suggests that **TBQQPt**-PDT may actively modulate the pathological ECM microenvironment of keloids, potentially facilitating long-term tissue remodeling. This aligns with the broader clinical goal of not only ablating the hyperactive cells but also normalizing the stiff, collagen-rich architecture of the scar to prevent recurrence.

These findings unveil a direct causal link between **TBQQPt**-mediated mitochondrial collapse and the suppression of keloid fibrosis. The biosynthesis and extracellular deposition of collagen by hyperactive HKFs is an exceptionally energy-demanding process. By triggering acute NADH depletion and mtDNA damage, **TBQQPt**-PDT fundamentally severs the cellular ATP supply. This bioenergetic crisis induces a state of 'energy starvation' that directly paralyzes collagen production and secretion. Concurrently, the localized oxidative stress drives intrinsic apoptosis to clear the pathogenic cells. This dual-pronged blockade, metabolic starvation at the biosynthetic level and programmatic clearance at the cellular level, effectively translates subcellular sabotage into the macro-level reduction of fibrotic collagen matrix.

## 3 Conclusion

In summary, we have engineered **TBQQPt**, a platinum (II)-coordinated donor-acceptor photosensitizer, to circumvent the intrinsic therapy resistance of keloid fibroblasts via a metabolism-interfering photodynamic strategy. The strategic integration of the Pt (II) center fundamentally reconfigures the excited-state dynamics of the organic chromophore, drastically amplifying both intersystem crossing and photoinduced electron-transfer efficiency. Diverging from conventional oxygen-constrained Type II photosensitizers, **TBQQPt** executes a synergistic Type I/Type II photodynamic mechanism, concurrently generating singlet oxygen and superoxide radicals while actively exhausting intracellular NADH. This Pt-driven electron extraction triggers an acute redox imbalance and mitochondrial metabolic collapse, effectively transforming conventional PDT into a dual-pronged oxidative and metabolic assault. Furthermore, **TBQQPt**'s preferential mitochondrial accumulation and high affinity for mitochondrial DNA facilitate localized ROS generation and direct genomic damage. This targeted sabotage of the mitochondrial apparatus ensures the induction of apoptosis even in the most resilient HKFs. Ultimately, this integrated photochemical–metabolic framework remains potent under the challenging hypoxic and fibrotic microenvironmental typical of pathological scars. Overall, this work establishes a metal-coordination-driven paradigm that extends photodynamic therapy beyond traditional ROS-centric frameworks toward metabolism-oriented photomedicine. The concept of Pt-assisted photoinduced redox catastrophe provides a robust blueprint for developing next-generation phototherapeutic agents to combat metabolically aberrant and therapy-resistant disorders.

## 4 Experimental section

All compounds utilized in this study were purchased from commercial vendors and utilized in their supplied form, with no further purification conducted. Beyotime was the supplier for the following materials: 3,3′-dioctadecyloxacarbocyanine perchlorate, pyridinium iodide, Calcein AM, Lyso-tracker Green, Mito-tracker Green, Mito-tracker Red CMXRos, CCK-8, and Hoechst 33342. Furthermore, the Annexin V-FITC/PI Apoptosis Detection Kit was obtained from Elabscience. All multi-channel images were acquired using the sequential scanning mode on the confocal microscope. The detection windows were configured with segregated bandpass filters: 580–620 nm for MitoTracker Red CMXRos and oxidized DHE, and 660–700 nm for the deep-red emission of **TBQQPt**.

*Synthesis of Compounds* ***1***: 4-(Diphenylamino) phenylboronic acid (2.89 g, 10 mmol) were added to a single-neck round-bottom flask, followed by the addition of 7-Bromobenzo[c][1,2,5]thiadiazole-4-carbaldehyde (2.43 g, 10 mmol) and potassium carbonate (K2CO3, 13.8 g, 10 mmol). Subsequently, tetrahydrofuran (THF, 25 mL) and methanol (50 mL) were added, and the catalyst 1,1′-bis(diphenylphosphino)ferrocene palladium (II) dichloride (Pd(dppf)Cl_2_, 10 mg) was introduced. The reaction mixture was stirred at 90°C for 6–8 h under a nitrogen atmosphere. After completion, the reaction was extracted with water and dichloromethane to remove excess potassium carbonate. The organic phase was concentrated under reduced pressure, and the crude product was purified by column chromatography to afford compounds **1**.

*Synthesis of Compounds* ***TBQB***: To a solution of compound **1** (203 mg, 0.5 mmol) in anhydrous toluene (5 mL), p-toluenesulfonic acid (172 mg, 1 mmol) and 4-Methylpyridine (47 mg, 0.5 mmol) were added. The mixture was heated to reflux under nitrogen for 12 h. The medium was concentrated and the resulting residue was dissolved in ethyl acetate (40 mL) and washed with water (50 mL). The organic layer was dried over anhydrous magnesium sulfate, filtered, and concentrated in vacuo. Purification on column chromatography (silica gel, methanol: dichloromethane 1:50, v/v) to afford a red solid (327 mg, 68%). ^1^H NMR (400 MHz, CDCl_3_) δ 8.63 (dd, *J* = 4.7, 1.4 Hz, 2H), 7.99 (d, *J* = 16.4 Hz, 1H), 7.93 – 7.85 (m, 1H), 7.84 – 7.75 (m, 1H), 7.70 (d, *J* = 7.5 Hz, 1H), 7.50 (dd, *J* = 4.8, 1.4 Hz, 1H), 7.34 – 7.27 (m, 2H), 7.24 – 7.15 (m, 3H), 7.12 – 7.03 (m, 1H). ^13^C NMR (101 MHz, CDCl_3_) δ 154.02, 153.93, 150.24, 148.36, 147.35, 144.91, 133.80, 130.40, 130.32, 130.00, 129.41, 129.03, 128.87, 127.62, 126.99, 125.04, 123.50, 122.64, 121.08, 77.36, 77.24, 77.04, 76.72. HR-MS (*m*/*z*): calculated for C_31_H_22_N_4_S [M+H]^+^, 483.1643; found, 483.1645.

*Synthesis of Compounds* ***TBQQ***: The synthesis of **TBQQ** was carried out following the same procedure as that used for **TBQB**. The organic layer was dried over anhydrous magnesium sulfate, filtered, and concentrated in vacuo. Purification on column chromatography (silica gel, methanol: dichloromethane 1:20, v/v) to afford a red solid (282 mg, 53%). ^1^H NMR (500 MHz, CDCl_3_) δ 8.95 (d, *J* = 8.2 Hz, 1H), 8.93 (d, *J* = 3.2 Hz, 1H), 8.36 (d, *J* = 7.8 Hz, 1H), 8.16 (d, *J* = 8.9 Hz, 1H), 7.95 – 7.87 (m, 2H), 7.81 – 7.75 (m, 2H), 7.75 – 7.68 (m, 3H), 7.63 (ddd, *J* = 8.2, 6.8, 1.2 Hz, 1H), 7.34 – 7.27 (m, 4H), 7.23 – 7.17 (m, 6H), 7.12 – 7.03 (m, 2H). ^13^C NMR (126 MHz, CDCl_3_) δ 154.16, 153.90, 150.09, 148.68, 148.40, 147.38, 143.38, 133.88, 131.00, 130.42, 130.05, 130.01, 129.66, 129.47, 129.42, 127.98, 127.71, 126.98, 126.70, 126.54, 125.06, 123.75, 123.52, 122.64, 116.71, 77.29, 77.03, 76.78. HR-MS (*m*/*z*): calculated for C_35_H_24_N_4_S [M+H]^+^, 533.1800; found, 533.1802.

*Synthesis of Compounds* ***TBQBPt***: Cisplatin (73 mg, 0.21 mmol) and AgNO_3_ (35 mg, 0.21 mmol) were stirred in anhydrous DMF (5 mL) for 24 h at room temperature in dark. After the removal of AgCl precipitate by filtration, the filtrate was added dropwise with **TBQB** (101 mg, 0.21 mmol) solution in DMF (5 mL), and the resulting mixture was stirred at 55^°^C for 48 h. Then DMF was removed in vacuo, and the residue was washed with CH_2_Cl_2_, acetone, and ether three times in sequence. The desired product **TBQBPt** (92 mg) was obtained as crimson solid in a yield of 59%. ^1^H NMR (500 MHz, DMSO-*d*_6_) δ 8.74 (d, *J* = 6.3 Hz, 2H), 8.14 (s, 1H), 8.06 (d, *J* = 7.5 Hz, 1H), 7.98 (d, *J* = 8.6 Hz, 1H), 7.92 (d, *J* = 7.4 Hz, 1H), 7.83 (d, *J* = 6.5 Hz, 1H), 7.36 (t, J = 7.8 Hz, 2H), 7.15 – 7.06 (m, 4H), 4.98 (s, 2H), 4.50 (s, 2H). ^13^C NMR (126 MHz, DMSO-*d*_6_) δ 153.74, 153.61, 153.28, 148.27, 147.27, 147.22, 133.70, 132.63, 131.41, 130.75, 130.34, 130.18, 128.72, 127.61, 127.20, 125.13, 124.21, 123.27, 122.42, 40.53, 40.36, 40.20, 40.03, 39.86, 39.69, 39.53, 21.23. HR-MS (*m*/*z*): calculated for C_31_H_28_N_6_PtS [M]^+^, 746.1427; found, 746.1423.

*Synthesis of Compounds* ***TBQQPt***: The synthesis of **TBQQ** was carried out following the same procedure as that used for **TBQBPt**. ^1^H NMR (500 MHz, DMSO-*d*_6_) δ 9.68 (d, *J* = 8.8 Hz, 1H), 9.28 (d, *J* = 5.7 Hz, 1H), 8.98 (d, J = 16.0 Hz, 1H), 8.60 (d, *J* = 8.5 Hz, 1H), 8.25 (dd, *J* = 15.6, 11.9 Hz, 2H), 8.16 – 8.06 (m, 2H), 8.02 (d, *J* = 8.6 Hz, 2H), 7.96 (d, *J* = 7.4 Hz, 1H), 7.89 (t, *J* = 7.6 Hz, 1H), 7.37 (t, *J* = 7.9 Hz, 4H), 7.15 – 7.08 (m, 8H), 4.65 (s, 3H), 4.47 (s, 3H). ^13^C NMR (126 MHz, DMSO-*d*_6_) δ 155.41, 153.81, 153.70, 148.33, 147.65, 147.23, 145.40, 133.95, 133.87, 131.44, 130.80, 130.35, 130.19, 128.73, 127.60, 127.40, 125.15, 124.53, 124.24, 122.44, 118.07, 40.53, 40.36, 40.20, 40.03, 39.86, 39.70, 39.53. HR-MS (*m*/*z*): calculated for C_35_H_30_N_6_PtS [M]^+^, 796.1584; found, 746.1576.

### 4.1 Cell culture and cell viability

Human keloid fibroblasts (HKFs) were obtained commercially (PSHX-C244I, Starfish-Bio) and maintained in HyCyte® Permanent Human Keloid Fibroblast Culture Medium (Starfish-Bio), under standard culture conditions (37°C, 5% CO_2_). Cells were passaged using trypsin upon reaching approximately 90% confluence. To evaluate cell viability, a Cell Counting Kit-8 assay (CCK-8; Dojindo, CK04) was performed in strict accordance with the manufacturer's instructions. Absorbance was measured using a Varioskan™ LUX microplate reader (Thermo Fisher Scientific, USA).

### 4.2 Intracellular reactive oxygen species detection

For the detection of intracellular reactive oxygen species, cells were digested with trypsin and seeded into 6-well plates at a density of 1 × 10^5^ cells per well, followed by incubation in a cell culture incubator. When cellular confluency reached 80%, the culture medium was aspirated, and the cells were rinsed three times with PBS. The cells were subsequently incubated with drug-containing treatment solution for 30 min, after which 10 μM DCFH-DA (a general ROS indicator) or 10 μM DHE (a superoxide anion-specific fluorescent probe) was added to each well, and incubation was continued in the dark for an additional 30 min. Following the removal of excess dye through PBS washes, the cells underwent designated treatments.

### 4.3 RNA-seq and enrichment analysis

Total RNA was extracted from collected cell or tissue samples with Trizol reagent according to the manufacturer's instructions. Eukaryotic mRNA was then purified using Oligo(dT) magnetic beads. After fragmenting the RNA, cDNA libraries were prepared and subjected to high-throughput sequencing on an Illumina NovaSeq 6000 system. Gene expression was calculated in fragments per kilobase of transcript per million mapped reads (FPKM). Differentially expressed genes (DEGs) were defined with an adjusted p-value <0.05 and |log_2_ fold change| ≥ 1.

For the resulting DEGs, Gene Ontology (GO) biological process enrichment analysis was conducted via the STRING database, limited to human annotations to pinpoint pathways enriched with common targets. Kyoto Encyclopedia of Genes and Genomes (KEGG) pathway enrichment results were graphically represented using the SangerBox platform (http://sangerbox.com).

### 4.4 Western blot

Cellular proteins were extracted using a RIPA lysis buffer supplemented with protease and phosphatase inhibitors. Subsequently, the proteins were separated by sodium dodecyl sulfate-polyacrylamide gel electrophoresis (SDS-PAGE) and transblotted onto PVDF membranes. Following transfer, membranes were blocked for 1 h at room temperature with 5% non-fat milk prepared in Tris-buffered saline containing 0.1% Tween-20 (TBST). Thereafter, membranes were incubated overnight at 4°C with the following primary antibodies: Bcl-2 (Proteintech, 80313-1-RR), Cleaved caspase-3 (Proteintech, 25128-1-AP), OXPHOS kit (Proteintech, PK30006) and GAPDH (Immunoway, PT0582R). After three washes with TBST, membranes were probed with horseradish peroxidase (HRP)-conjugated secondary antibodies. Immunoreactive bands were detected using an enhanced chemiluminescence kit (E− Elabscience, BC-K318-M), visualized on a ChemiDoc Touch imaging system (Bio-Rad, USA), and quantified using ImageJ software.

### 4.5 Patient-derived xenograft keloid-implantation mouse model

This keloid mouse model was developed by modifying an established xenograft protocol [36]. Twenty female BALB/c nude mice (3 w) were obtained from Hunan SJA Laboratory Animal Co., Ltd and housed under specific pathogen-free conditions. The animal experimental protocol was approved by the Animal Ethics Committee of Central South University (No. APU-2025-0386). Keloid tissue samples were collected from three patients at the Department of Dermatology, Third Xiangya Hospital, Central South University, with ethical approval (No. 2025-S923). The specimens were immediately placed in serum-free DMEM, stored at 4°C, and transported to the animal facility. After dissecting away epithelial, adipose, and necrotic tissue, the samples were rinsed three times with DPBS and cut into uniform 4 × 4 × 5 mm^3^ pieces under sterile conditions. Each piece was implanted subcutaneously into the upper back of a mouse, and the wound was sutured with 6-0 stitches. Following a 7-day recovery period, the animals with established keloid grafts were used for treatment experiments.

### 4.6 Treatment

Mice bearing successfully established keloid xenografts were assigned to four experimental groups (n = 5) using a computer-generated random number table to eliminate allocation bias: control (G1), photosensitizer alone (G2), light irradiation alone (G3), or combined photosensitizer and irradiation treatment (G4). Prior to each light exposure, animals were anesthetized using inhaled isoflurane. Irradiation was performed with the light source positioned perpendicularly 20–30 cm above the graft site for a duration of 10 min per session. Daily, before treatment, individual mouse body weight was recorded, and the longest (L) and shortest orthogonal diameters (S) of the subcutaneous implant were measured with calipers. Graft volume was estimated using formula V = 0.5 × L × S^2^. On day 9 post-intervention, all mice were euthanized, and keloid xenografts were harvested surgically for subsequent analysis.

### 4.7 Statistical analysis

All data were obtained from at least three independent experiments and were expressed as mean ± SD. The exact sample size for each experimental group is shown in every Figure as the number of dots. Two-tailed unpaired Student's t-test was used to compare the differences between two different treatment groups, and one-way ANOVA with post hoc tests was used to compare the differences among the three or more different treatment groups. All statistical analyses were accomplished using GraphPad Prism software (v 9.3). Significance levels were denoted as follows: ∗*p* < 0.05, *p* < 0.01, ∗*p* < 0.001, and ns (not significant).

## CRediT authorship contribution statement

**Fugang Xiao:** Data curation, Formal analysis, Methodology, Software, Visualization, Writing – original draft. **Xiang Cheng:** Data curation, Formal analysis, Software, Visualization, Writing – original draft. **Jianbo Chen:** Formal analysis, Software. **Yanpeng Fang:** Methodology, Software. **Xingru Zhou:** Formal analysis. **Conghui Liu:** Data curation. **Zhibing Fu:** Methodology. **Ningling Wu:** Writing – review & editing. **Yifei Xie:** Supervision. **Lu Zhou:** Writing – review & editing. **Shenming Xu:** Investigation. **Wenbing Zeng:** Conceptualization, Funding acquisition, Investigation, Project administration, Resources, Supervision, Validation, Visualization, Writing – review & editing. **Jinrong Zeng:** Conceptualization, Funding acquisition, Investigation, Project administration, Visualization, Writing – original draft, Writing – review & editing.

## Declaration of competing interest

The authors declare that they have no known competing financial interests or personal relationships that could have appeared to influence the work reported in this paper.

## Data Availability

Data will be made available on request.
