# Supplementary material for: Mitochondria-targeted phototherapeutic system enabling spatiotemporal-controlled NADH depletion for keloid intervention
Source: Mater Today Bio. 2026 Jun 30;39:103427. doi: 10.1016/j.mtbio.2026.103427 (PMC13355567; doi:10.1016/j.mtbio.2026.103427)
Supplement: Multimedia component 1 [file mmc1.docx]

# **Experimental Procedures**

**Materials and methods**

***AIE Property***

The AIE properties of the PSs were evaluated by measuring their fluorescence spectra in DMSO/PBS mixtures with varying volume ratios (10 μM PS per sample).

***Overall ROS Detections***

DCFH was utilized to detect the ROS generation of PSs in aqueous solution under white light irradiation. DCFH was converted from DCFH-DA (0.5 mL, 1 mM in ethanol) reacting with an aqueous solution of NaOH (2 mL, 10 mM) for 30 min at room temperature. The hydrolysate was then neutralized with 7.5 mL of PBS buffer solution to get the stock solution with a concentration of 50 μM. PBS buffer solution containing 5 μM DCFH was mixed separately with different samples. The fluorescence of PS sensitized DCFH under white light irradiation (20 mW cm^−2^) was measured at different time intervals. The PL spectra were measured with excitation at 488 nm and emission was collected from 500 to 600 nm. The enhancement of DCFH fluorescence intensity (I/I0) at 530 nm was used to indicate the total ROS generation.

***Singlet Oxygen (^1^O_2_) Detection***

^1^O_2_ generation was monitored using 9,10-anthracenediyl-bis (methylene) dimalonic acid (ABDA) as the chemical trap. A mixture of PBS buffer (containing 50 μM ABDA from a 10 mM DMSO stock solution) and 10 μM PS was irradiated with light (20 mW cm^-^²) for varying durations. The ^1^O_2_ production was quantified by measuring the decrease in ABDA absorbance at 378 nm and expressed as the ratio of absorbance after irradiation to initial absorbance (A-A_0_).

***Superoxide Radical (•O_2_^−^) Detection (PDT)***

To assess superoxide radical (•O_2_^−^) generation, a PBS solution containing 10 μM Dihydrorhodamine 123 (DHR 123, from a 2 mM DMSO stock) was mixed with different PS samples (final concentration: 10 μM each). The mixture was irradiated with light (20 mW cm^-^²). The FL spectra was collected from 530 to 700 nm. The enhancement of DHR123 fluorescence intensity (I/I_0_) at 526 nm was used to indicate the •O_2_^−^ generation.

***Hydroxyl Radical (•OH) Detection***

•OH generation was detected using hydroxyphenyl fluorescein (HPF) as the fluorescent probe. A PBS solution containing 10 μM HPF (from a 5 mM DMF stock solution) and 5 μM PS was irradiated with light (20 mW cm^-2^) for varying durations. Fluorescence spectra (λex = 480 nm, λem = 500-650 nm) were recorded, and •OH production was quantified by the fluorescence intensity ratio at 516 nm (I/I_0_).

***Photocatalytic Oxidation of NADH***

PBS buffer solution containing 200 μM NADH was mixed with PSs (10 μM). The UV-vis spectra were recorded after each light irradiation (20 mW cm^-2^) period. The absorbance of NADH at 340 nm declines indicate the photooxidation of NADH.

***ESR Analysis***

The type of the produced ROS were further characterized by ESR spectroscopy using specific spin-trapping agents. For ^1^O_2_ detection, 100 μL DMSO solution containing 50 mM TEMP was mixed with PS. After light irradiation (20 mW cm^-^², 5 min), the solution was transferred to a quartz capillary tube for ESR measurement. •O_2_^−^ was trapped using 100 μL DMSO solution containing 50 mM DMPO mixed with PS, followed by immediate ESR analysis. •OH was similarly detected in PBS buffer (100 μL) containing 50 mM DMPO and PS. All ESR spectra were recorded at room temperature using standard spectrometer parameters.

***Theoretical Calculation***

The initial molecular structure of the compound was generated using the Molecular Operating Environment (MOE). For subsequent analysis, density functional theory (DFT) was employed to perform geometry optimization at the B3LYP/6-311G(d,p) level. Time-dependent DFT (TD-DFT) was used to calculate the excited states at the CAM-B3LYP/6-311G(d,p) level. The Becke–Johnson damping (DFT-D3(BJ)) correction was applied to account for dispersion effects. The solvent effect of water was incorporated using the solvation model based on density (SMD). All DFT calculations were carried out using Gaussian 16. Orbital visualization was achieved with the help of Multiwfn 3.8 and VMD software.

***Scratch Wound Healing Assay***

HKFs cells were seeded in 6-well plates at 4 ×10⁵ cells per well. After 24 h, a scratch assay was performed by introducing a linear wound using a 20-μl pipette tip. Images were collected by microscope (Nikon) at different time points. The collection of images was discontinued once the scratch wounds in the negative control group had completely healed.

***Quantitative Real-Time PCR (qRT-PCR)***

Total RNA was isolated from harvested cells or tissues using Trizol following the manufacturer's protocol. cDNA was synthesized using the High-Capacity cDNA Reverse Transcription Kit (Invitrogen). Quantitative PCR was performed using 2X SYBR Green qPCR Master Mix (Accurate-Bio, AG11701) following the manufacturer’s guidelines. GAPDH was used as the internal control. Primers were bought from Tsingke Biotech and the sequences of primers were as follows: - Human Actin: CATGTACGTTGCTATCCAGGC (forward, 5′−3′), CTCCTTAATGTCACGCACGAT (reverse, 5′−3′); - Human mt-ND1: CTCTTCGTCTGATCCGTCCT (forward, 5′−3′), TGAGGTTGCGGTCTGTTAGT (reverse, 5′−3′); - Human mt-Dloop: CATCTGGTTCCTACTTCAGGG (forward, 5′−3′), CCGTGAGTGGTTAATAGGGTG (reverse, 5′−3′).

***Apoptosis Detection***

Following intervention, cells were maintained in an incubator at 37 °C and 5% CO₂ for 24 h before apoptosis detection. Cells were digested by trypsin solution without EDTA (Procell, PB180228) and rinsed by cold DPBS. Annexin V-FITC/PI Apoptosis Detection Kit (Elabscience , E-CK-A211) was applied according to the manufacturer’s protocol.

***Immunofluorescence and immunohistochemical staining***

HKFs were plated in 12-well plates at a density of 1–2 × 10⁵ cells per well and allowed to adhere overnight under standard culture conditions (37°C, 5% CO₂). Following the designated treatment, cells were rinsed twice with PBS and fixed using 4% paraformaldehyde (Servicebio, G1101) for 15 minutes at room temperature. Subsequently, cell membranes were permeabilized with 0.5% Triton X-100 (Beyotime, P0096) for 10 minutes, followed by blocking with 5% bovine serum albumin (BSA; Beyotime, ST2254) in PBS for 1 hour. Incubation with primary antibodies targeting 8‑OHdG (MCE, HY-P81140) and TOMM20 (Abcam, ab56783) was carried out at 4°C overnight. After washing three times with PBS, samples were exposed to fluorescent secondary antibodies (Alexa Fluor® 594, ab150080; Alexa Fluor® 488, ab150113) for 1 hour in the dark. A final PBS wash preceded nuclear counterstaining with DAPI-containing mounting medium (Solarbio, S2110) for 10 minutes. Fluorescence images were captured using a Nikon fluorescence microscope.

Tissue samples were fixed in 4% paraformaldehyde at 4°C for 18–24 hours and then paraffin-embedded. Consecutive 5 μm sections were cut, deparaffinized, and rehydrated. Antigen retrieval was performed using heated sodium citrate buffer (pH 6.0). After blocking endogenous peroxidase and nonspecific binding, sections were incubated overnight at 4°C with primary antibodies against PCNA (Selleck, F0018) or cleaved caspase-3 (Affinity, AF7022). Subsequently, sections were incubated with corresponding HRP-conjugated secondary antibodies at room temperature, followed by DAB chromogenic development. Finally, sections were counterstained with hematoxylin, dehydrated, cleared, and mounted with neutral balsam.

***H&E Staining***

Tissue sections were dewaxed with environment-friendly dewaxing reagent (Solarbio, YA0031) and rehydrated with ethanol at different concentrations, and then they were stained with hematoxylin for 3 min and rinsed with running water. After that, they were stained with eosin for 10 s (Solarbio, G1120).

***Intracellular NAD^+^/NADH Assay***

Intracellular NAD^+^ and NADH levels were quantified using an NAD^+^/NADH Colorimetric Assay Kit (E-BC-K804-M, Elabscience). Briefly, treated HKFs were harvested and homogenized in 0.4 ml of pre-cooled Extracting Solution, followed by centrifugation at 12,000g for 10min at 4°C. The supernatant was passed through a 10 KD ultrafiltration tube to remove endogenous enzymes. Total NAD was measured directly from the filtrate. For individual NADH quantification, the filtrate was heated at 60°C for 30 min to decompose NAD^+^. After adding the reaction working solution and chromogenic agent, the absorbance was measured at 450nm. Data were normalized against total protein concentrations determined by a BCA Protein Assay Kit.

***Hemolysis Assay***

The hemocompatibility of **TBQQPt** was evaluated using a standard in vitro hemolysis assay. Fresh mice red blood cells (RBCs) were collected, washed multiple times with PBS, and diluted to form a 2% (v/v) RBC suspension. Subsequently, the RBC suspension was mixed with various concentrations of **TBQQPt** to yield final drug concentrations ranging from 5 to 400 µM. PBS and deionized water were used as negative and positive controls, respectively. After incubation at 37 °C for 2 h, the mixtures were centrifuged (1000 × g, 5 min). The supernatants were collected, and the absorbance of released hemoglobin was measured at 540 nm using a microplate reader. The hemolysis ratio was calculated according to the formula: Hemolysis (%) = (A_sample_ - A_negative_)/ (A_positive_ - A_negative_)×100%.

***Serum Biochemistry and Cytokine Analysis***

At the end of the in vivo treatments, blood samples were collected from the mice and centrifuged to obtain the serum. The serum activities of alanine aminotransferase (ALT) and aspartate aminotransferase (AST), as well as the concentrations of creatinine (CRE) and blood urea nitrogen (BUN), were quantitatively determined using the Amplex Red ALT Activity Assay Kit (P2711S, Beyotime), Amplex Red AST Activity Assay Kit (P2715S, Beyotime), Amplex Red Creatinine Assay Kit (S0291S, Beyotime), and Urea Assay Kit (S0574S, Beyotime), respectively, in strict accordance with the manufacturer's protocols. The systemic inflammatory response was evaluated by quantifying the serum concentration of TNF-α using a Mouse TNF-α ELISA Kit (E-EL-M3063, Elabscience).

***DNA Photocleavage Assay***

The DNA photocleavage activity of **TBQQPt** was evaluated using pUC-19 plasmid DNA (2686 bp) (8200025, Simgen). Briefly, the plasmid DNA was mixed with different concentrations of **TBQQPt** (0, 5, 10, 20, and 50 µM) in Tris-HCl buffer solution (10 mM, pH 7.4, diluted from 1 M stock, Solarbio). The mixtures were then exposed to white light irradiation (20 mW cm⁻²) for 5 min. After irradiation, each sample was mixed with 6× DNA Loading Buffer (Solarbio). Electrophoresis was performed on a 1% (w/v) agarose gel (Solarbio) prepared in 1× TAE buffer (Solarbio) containing Super GelRed II nucleic acid stain (Xinjing Biotech). The gel was run at a constant voltage in 1× TAE running buffer. A DL5000 DNA Ladder (100 to 5,000 bp, Xinjing Biotech) was utilized as the molecular weight marker. Finally, the DNA bands were visualized and photographed under a UV transilluminator gel imaging system.

# **Supporting Experiments**

**Scheme S1**. The synthetic routes for **TBQB**, **TBQQ**, **TBQBPt** and **TBQQPt**.


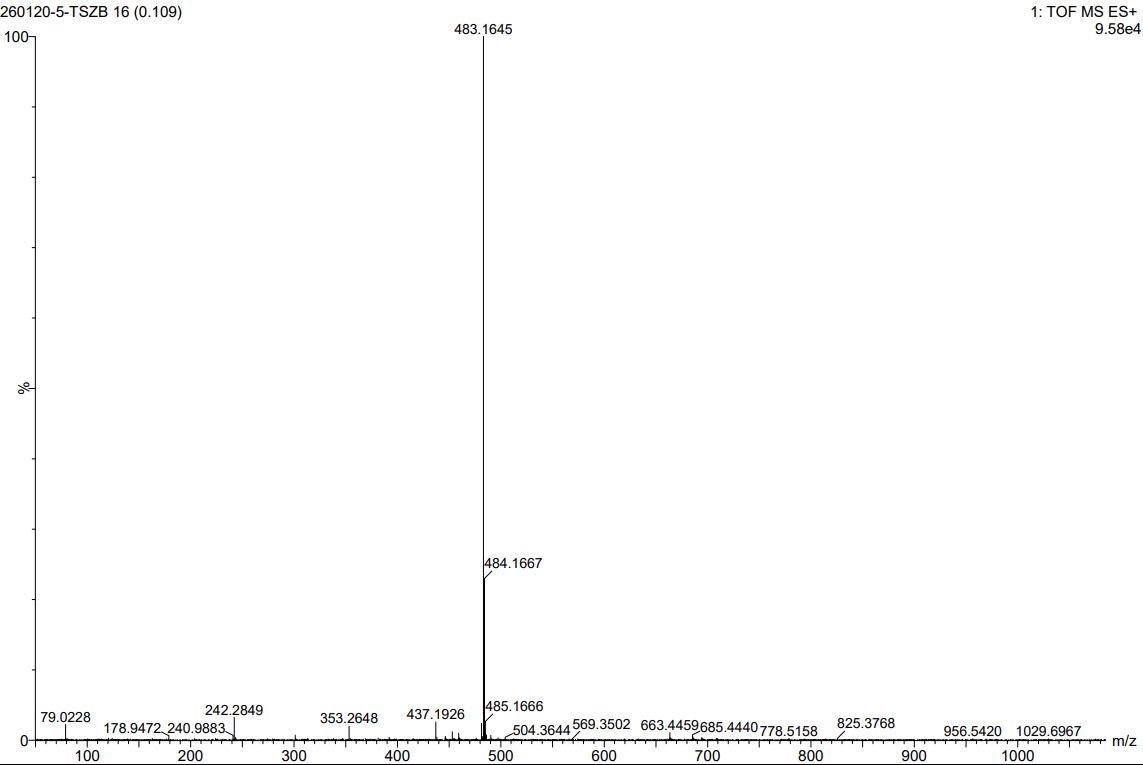


**Figure S1.** HRMS spectra of compound **TBQB**.


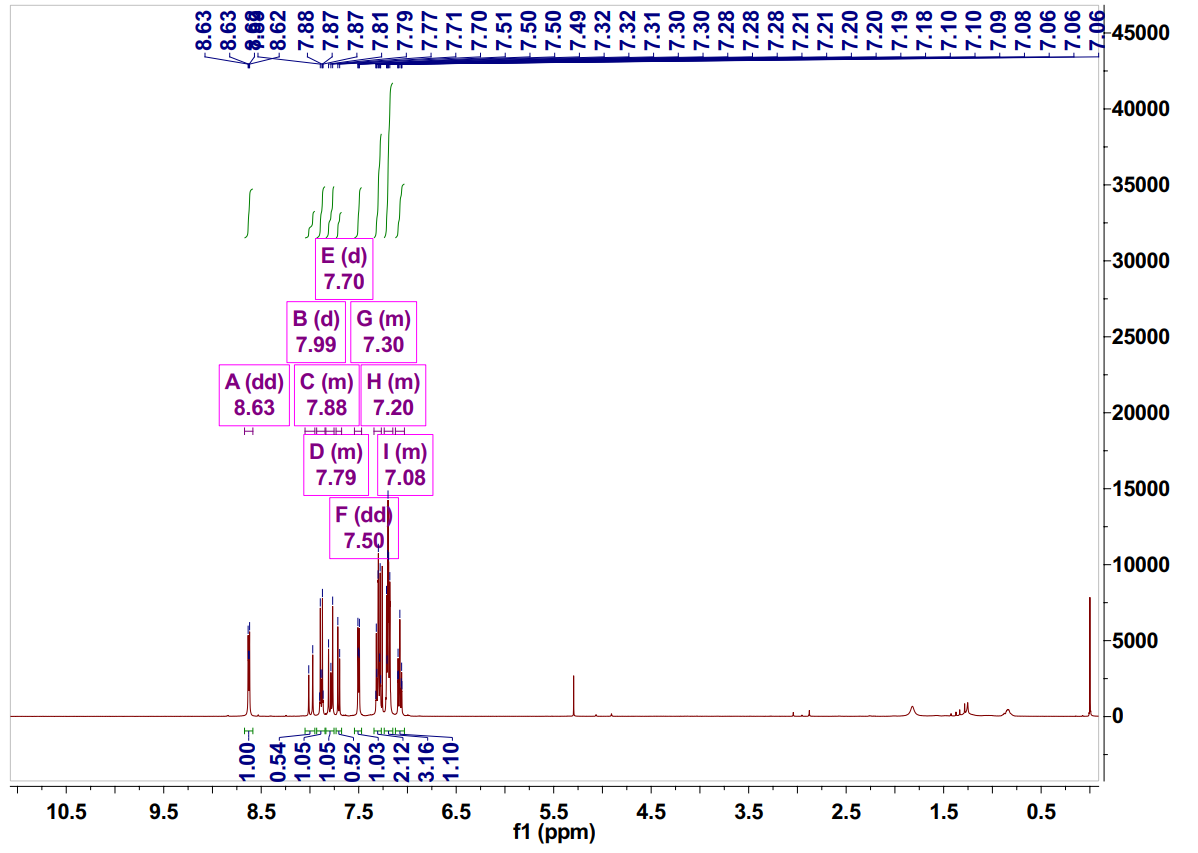


**Figure S2.** ^1^H-NMR spectra of compound **TBQB** in CDCl_3_.


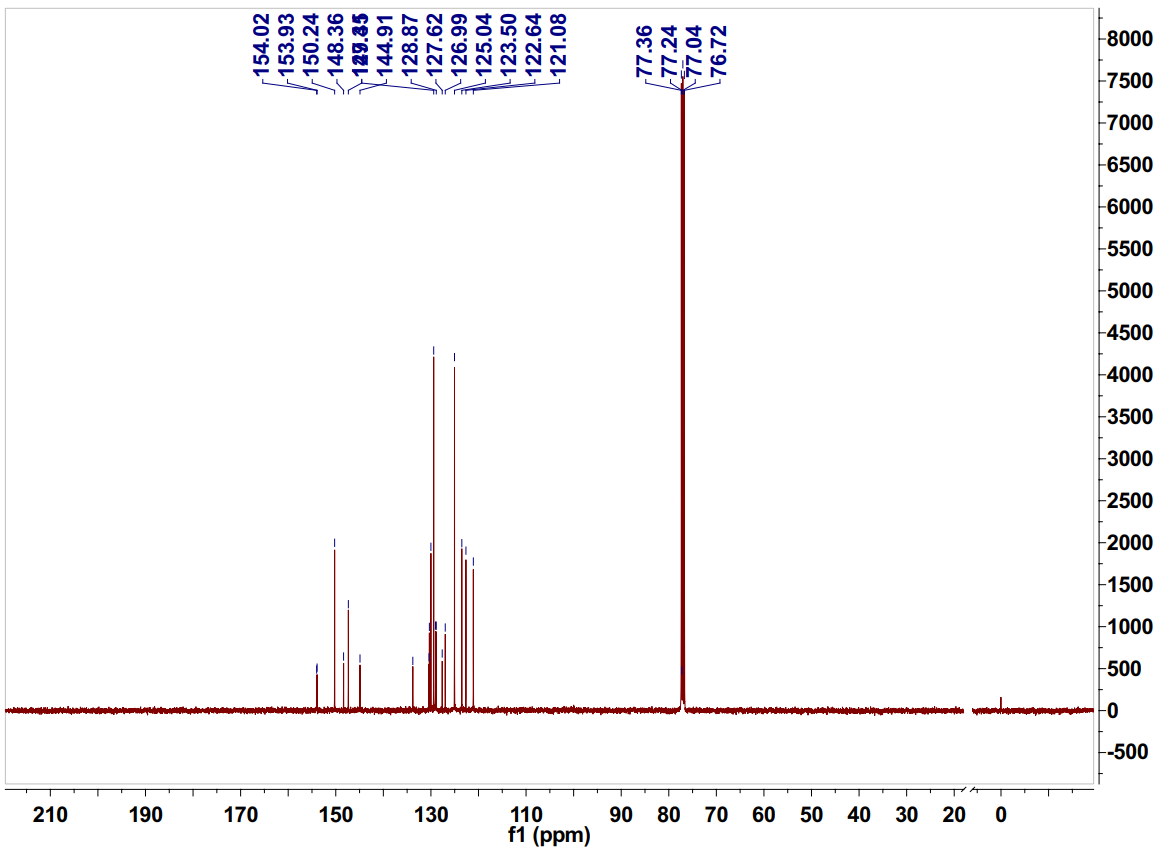


**Figure S3**. ^13^C-NMR spectra of compound **TBQB** in CDCl_3_.


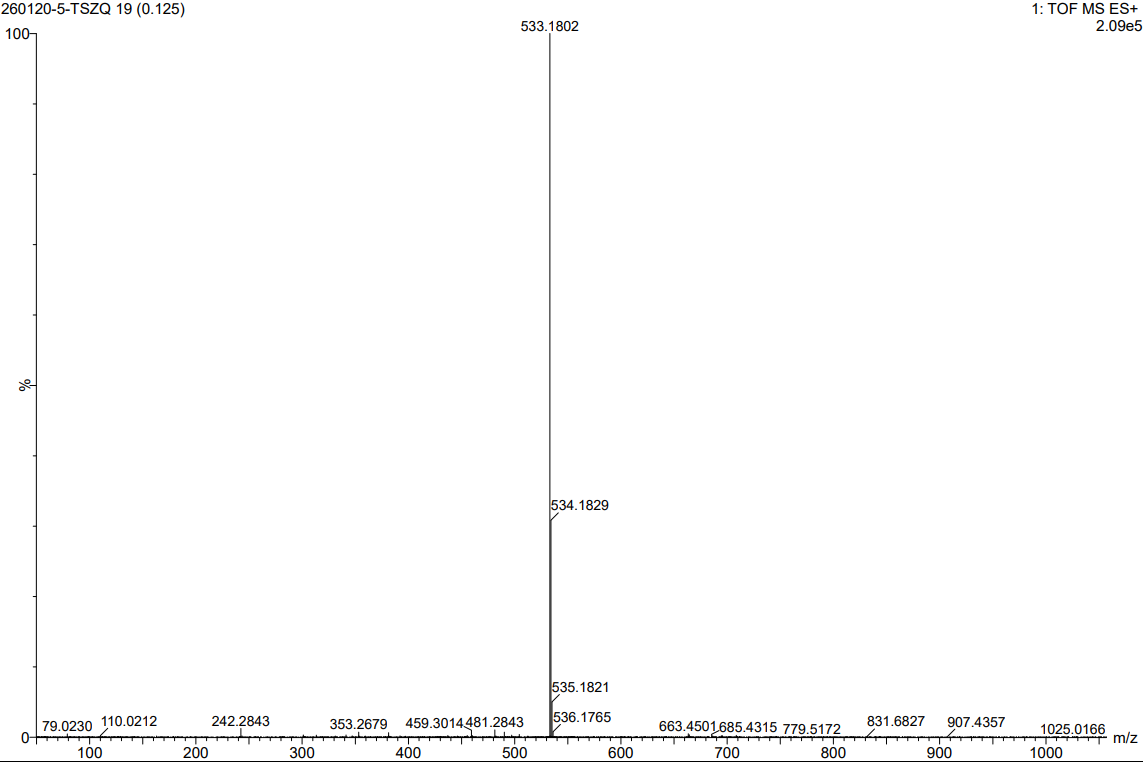


**Figure S4.** HRMS spectra of compound **TBQQ**.


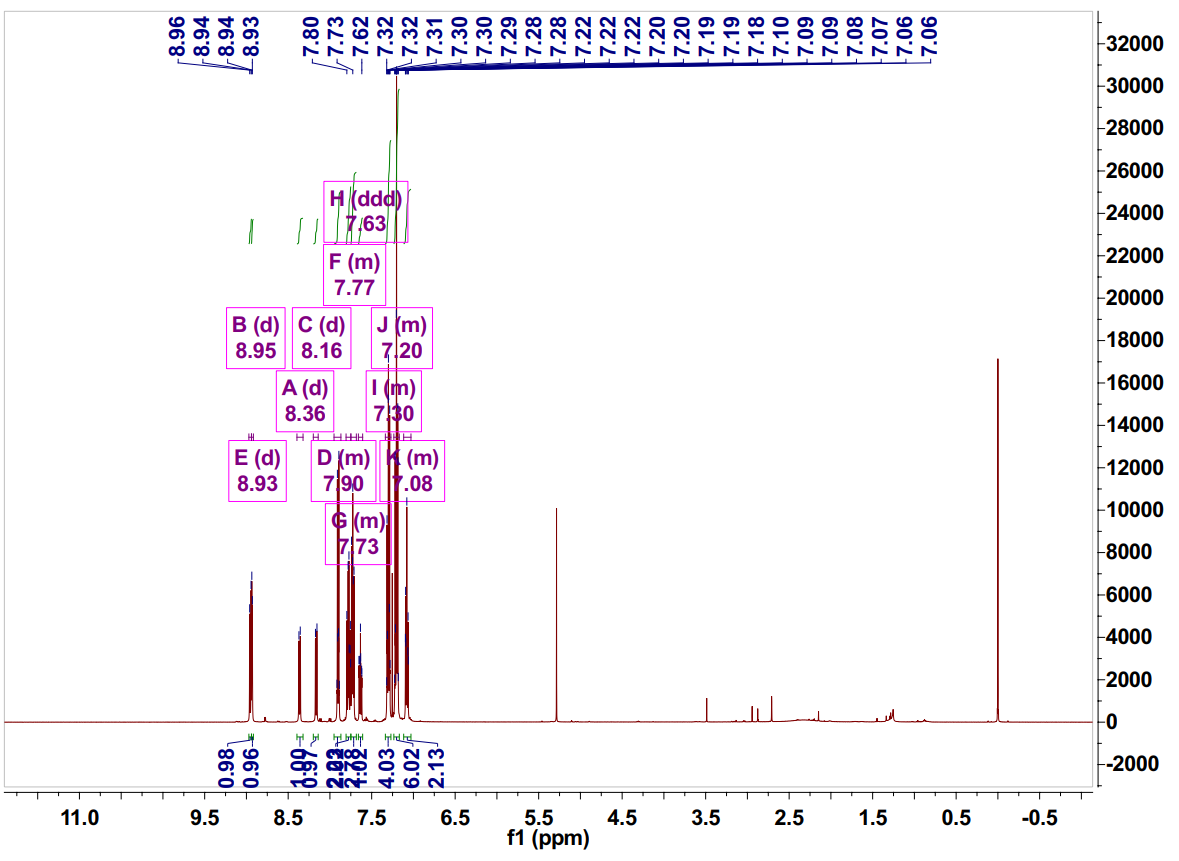


**Figure S5.** ^1^H-NMR spectra of compound **TBQQ** in CDCl_3_.


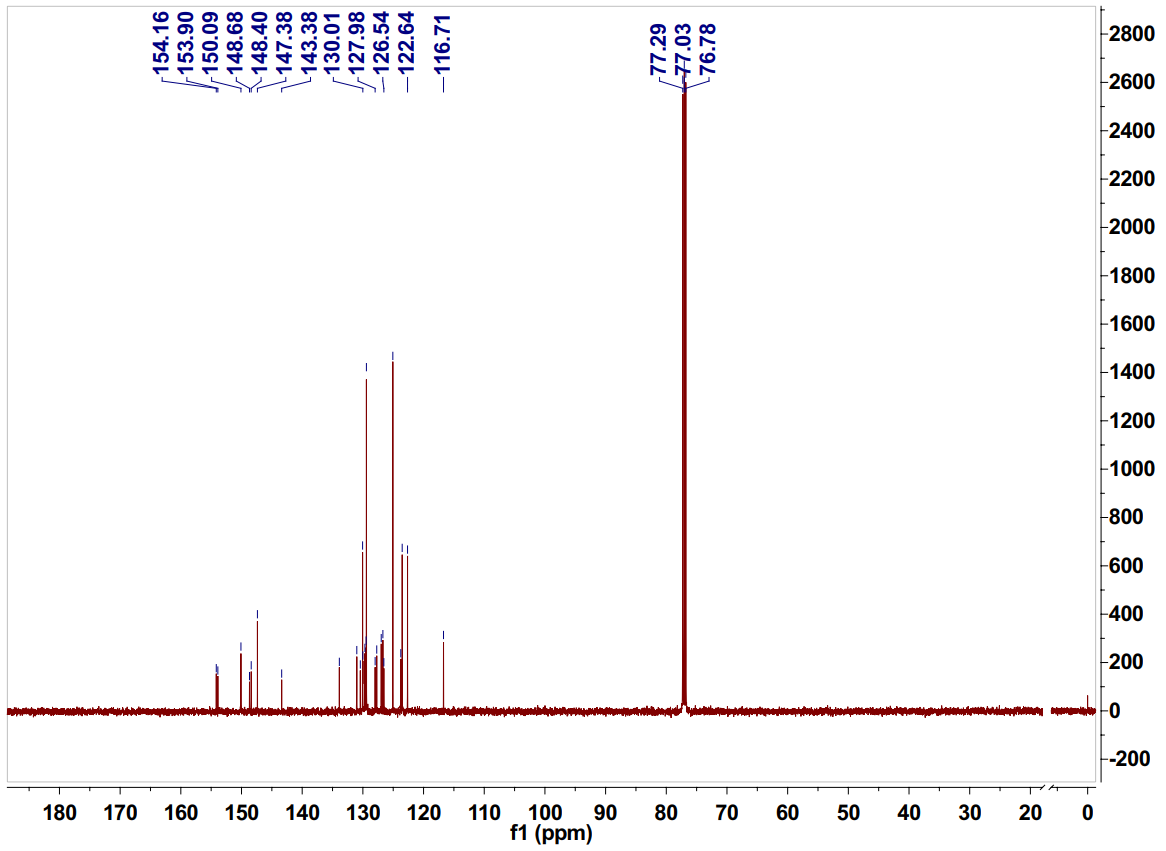


**Figure S6**. ^13^C-NMR spectra of compound **TBQQ** in CDCl_3_.


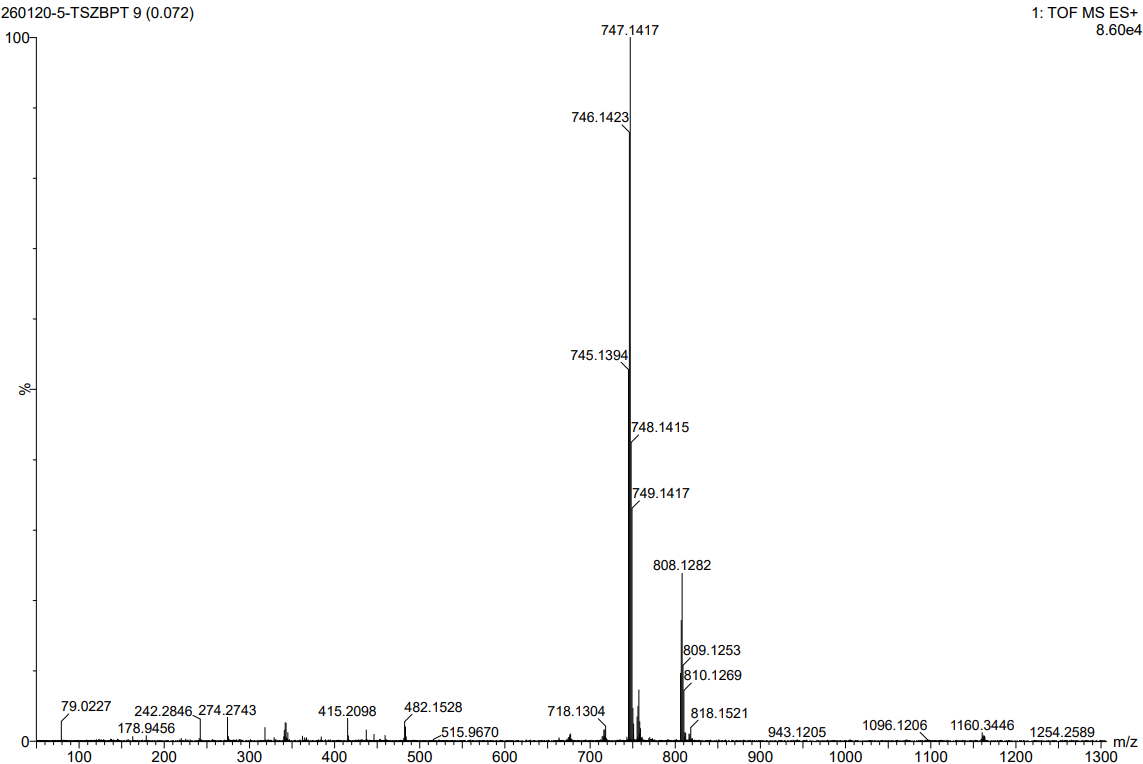


**Figure S7.** HRMS spectra of compound **TBQBPt**.


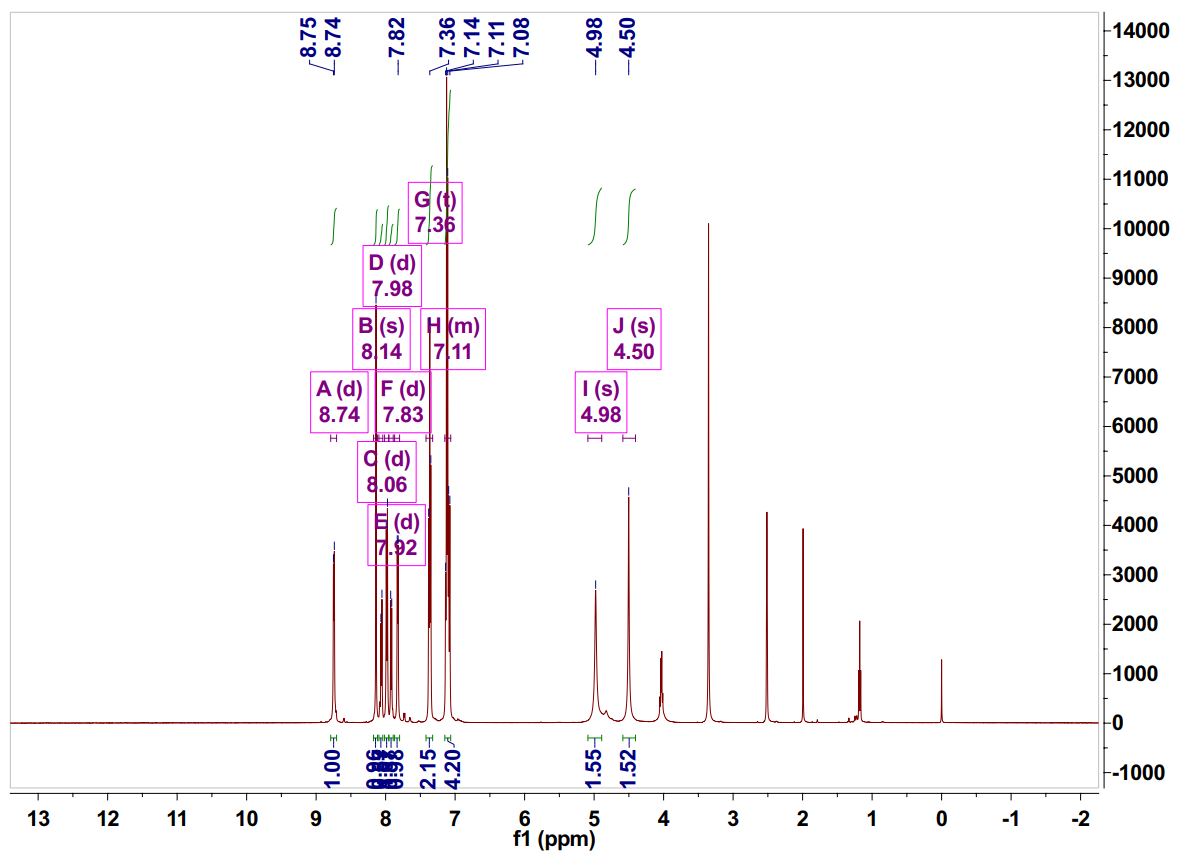


**Figure S8.** ^1^H-NMR spectra of compound **TBQBPt** in DMSO-*d6*.


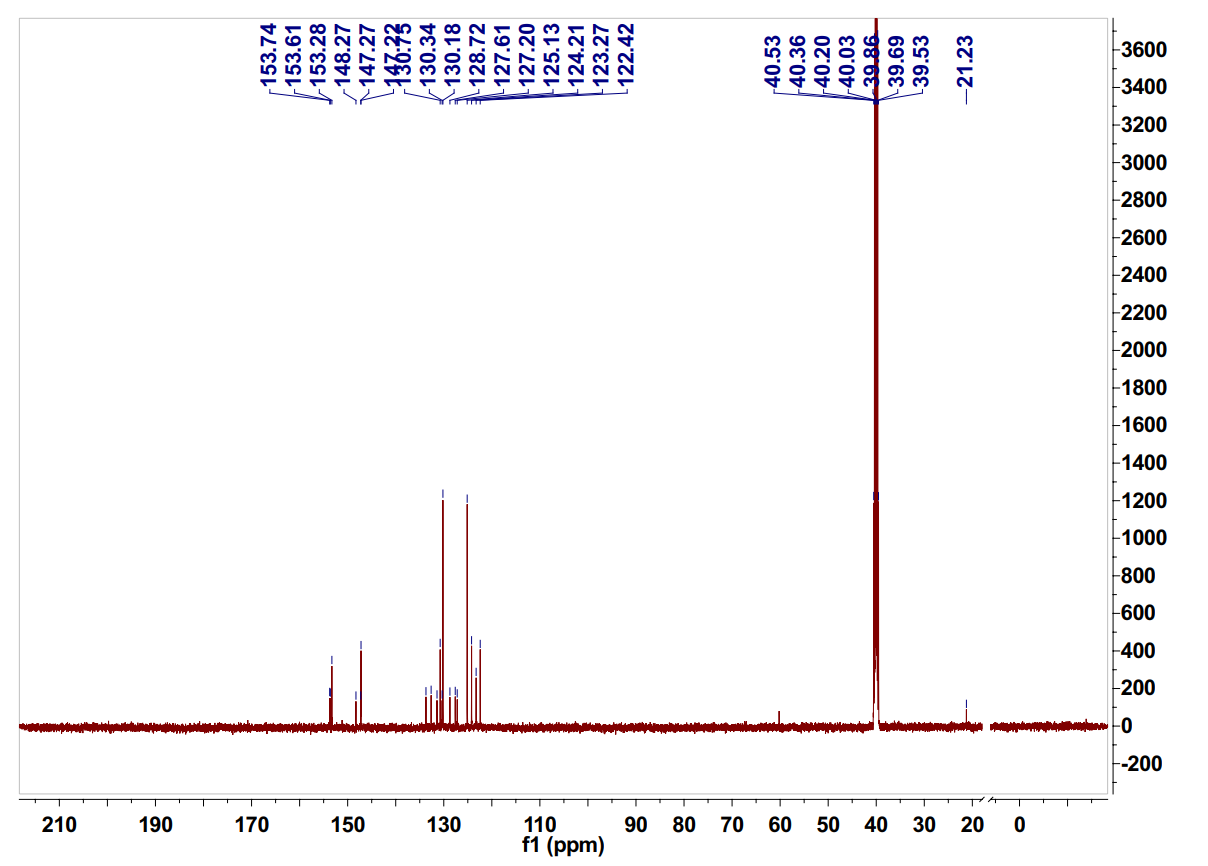


**Figure S9.** ^13^C-NMR spectra of compound **TBQBPt** in DMSO-*d6*.


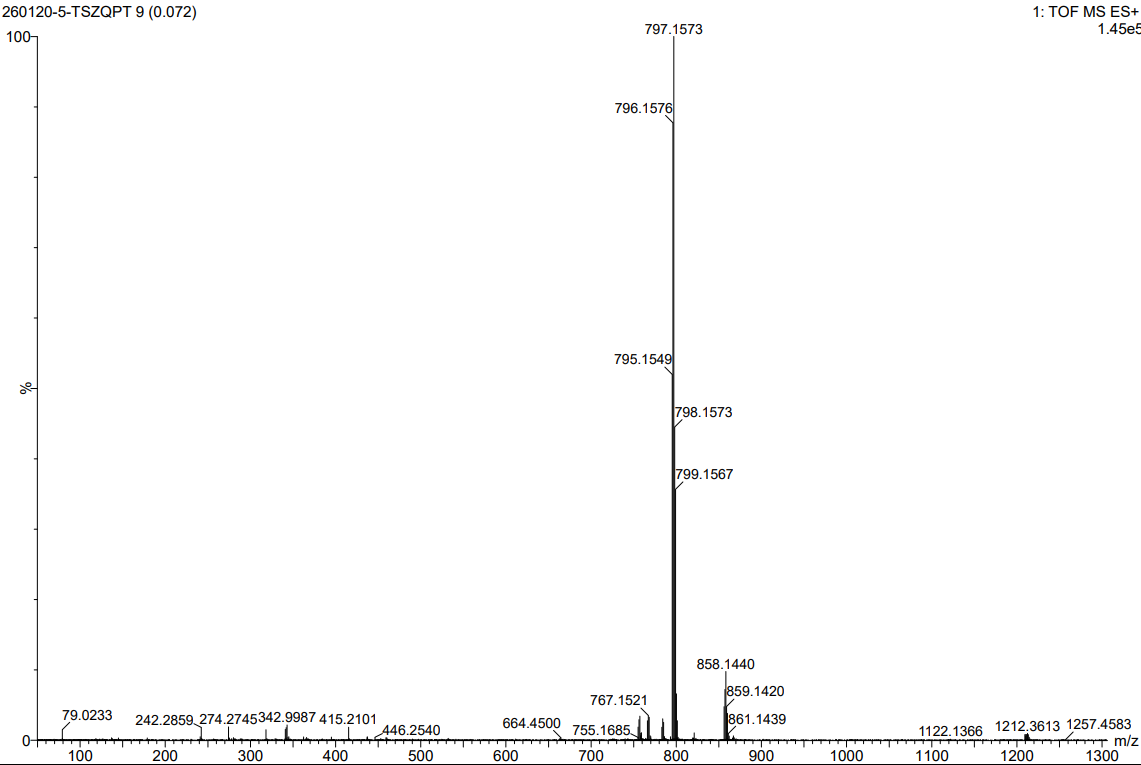


**Figure S10.** HRMS spectra of compound **TBQQPt**.


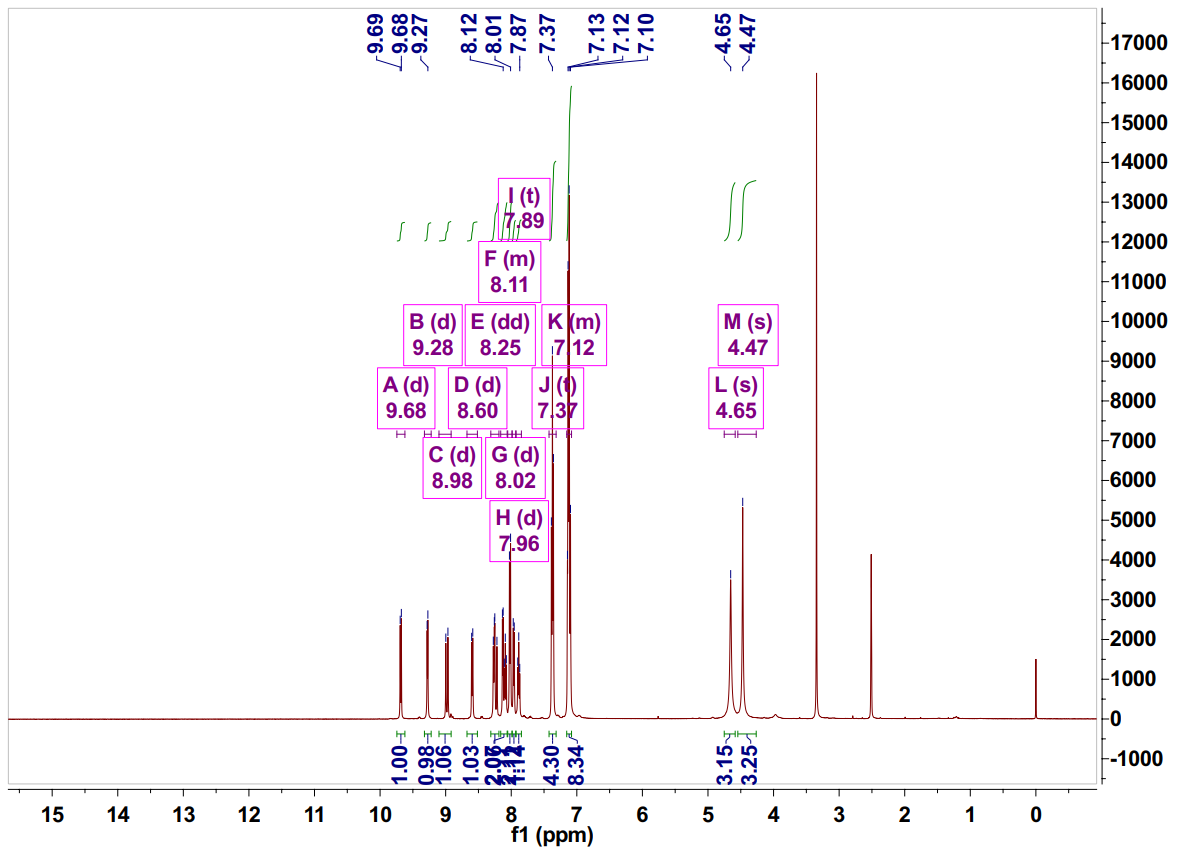


**Figure S11.** ^1^H-NMR spectra of compound **TBQQPt** in DMSO-*d6*.


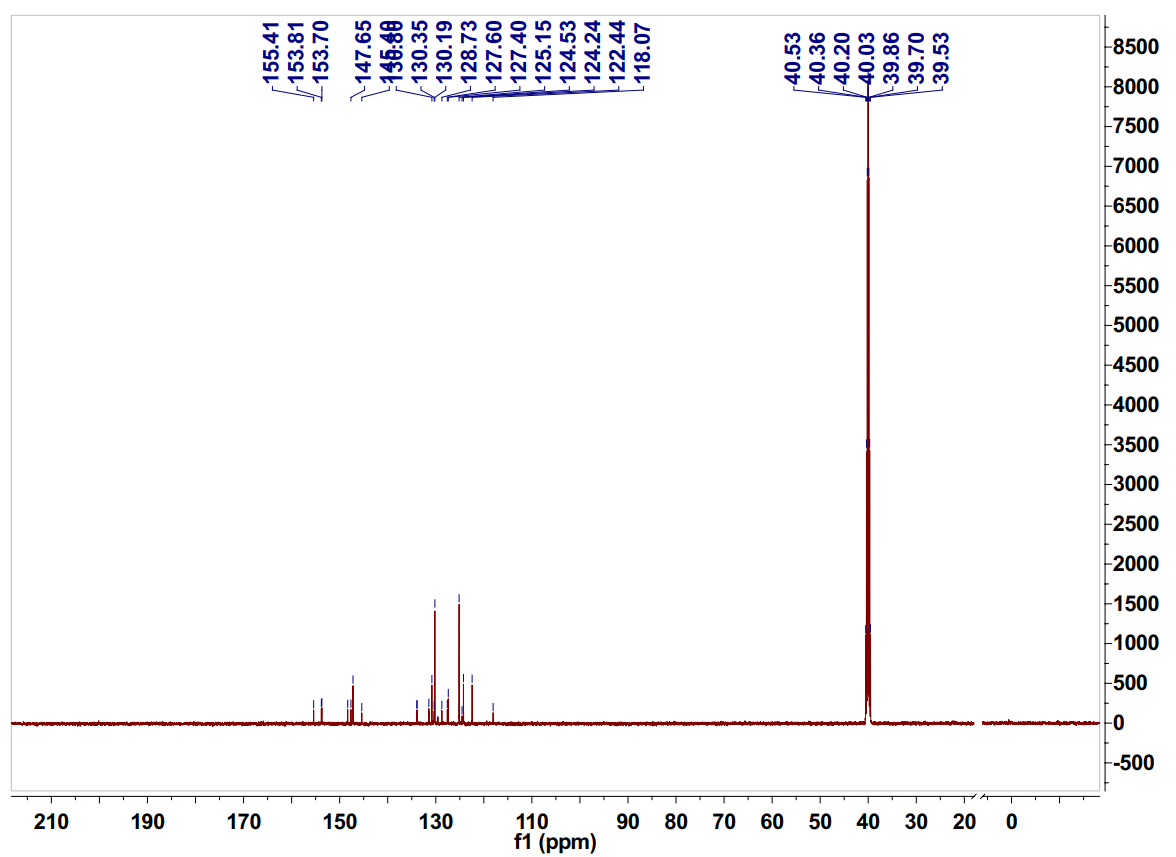


**Figure S12.** ^13^C-NMR spectra of compound **TBQQPt** in DMSO-*d6*.


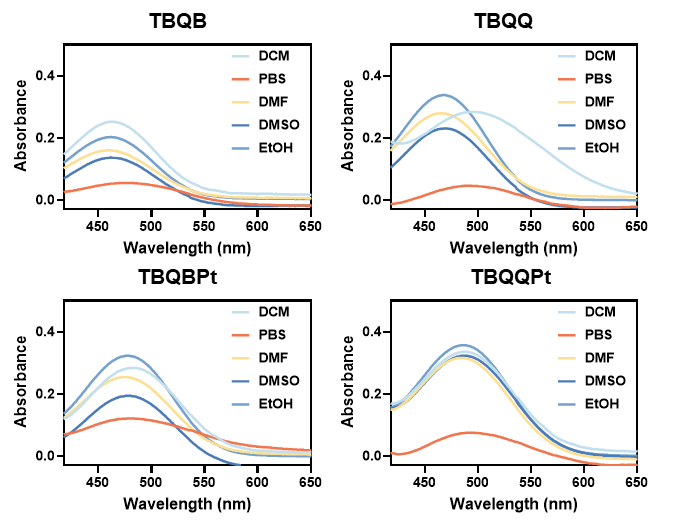


**Figure S13.** UV–Vis absorption spectra of **TBQB, TBQQ, TBQBPt and TBQQPt** in DCM, PBS, DMF, DMSO and EtOH containing 1% DMSO at a concentration of 10 μM.


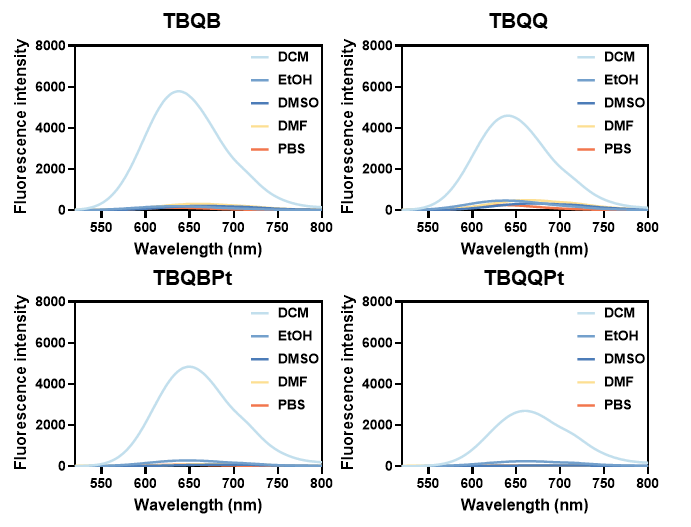


**Figure S14.** Fluorescence emission spectra of **TBQB, TBQQ, TBQBPt and TBQQPt** in DCM, PBS, DMF, DMSO and EtOH containing 1% DMSO at a concentration of 10 μM.


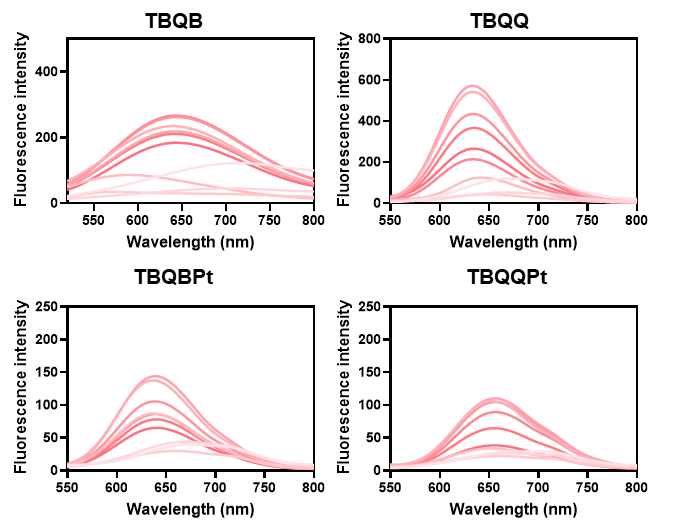


**Figure S15.** Fluorescence emission spectra of **TBQB, TBQQ, TBQBPt and TBQQPt** in DMSO/PBS mixtures with PBS fractions ranging from 0% to 99%.


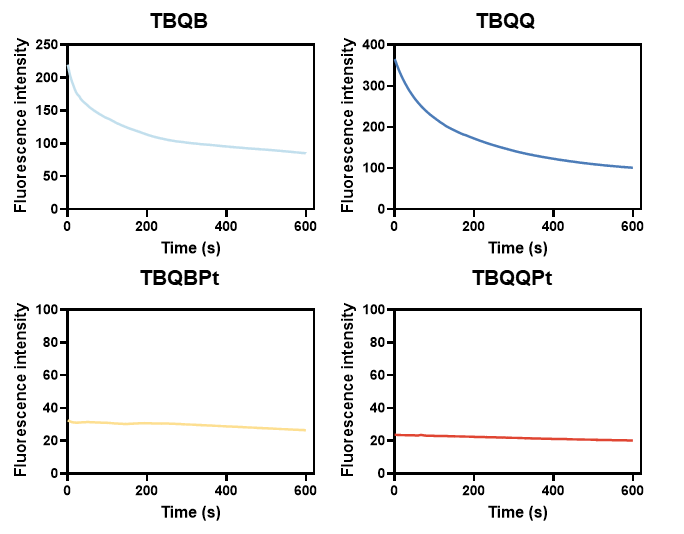


**Figure S16.** Photostability of PSs under continuous light irradiation, monitored by FL intensity over time.


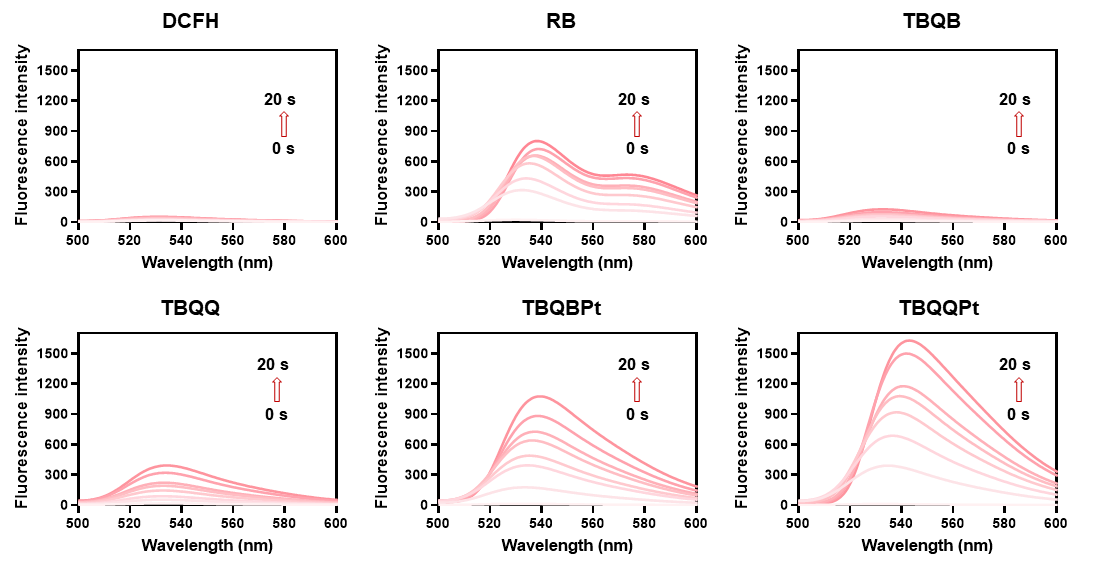


**Figure S17.** FL spectra of DCFH in PBS in present of **TBQB, TBQQ, TBQBPt, TBQQPt**, **RB** and PBS under white light irradiation.


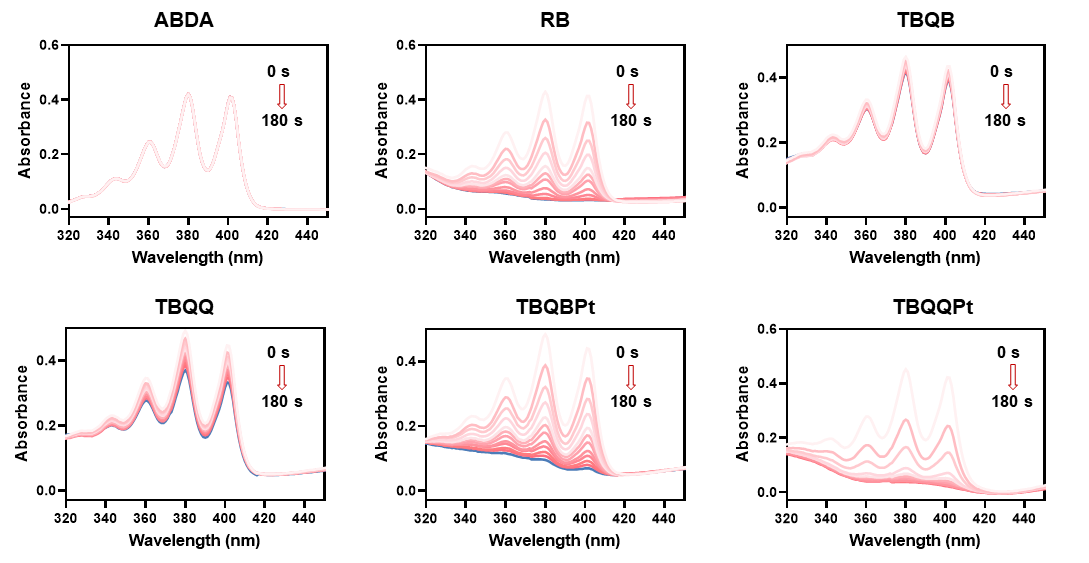


**Figure S18.** UV-vis spectra of ABDA in PBS in present of **TBQB, TBQQ, TBQBPt, TBQQPt**, **RB** and PBS under white light irradiation.


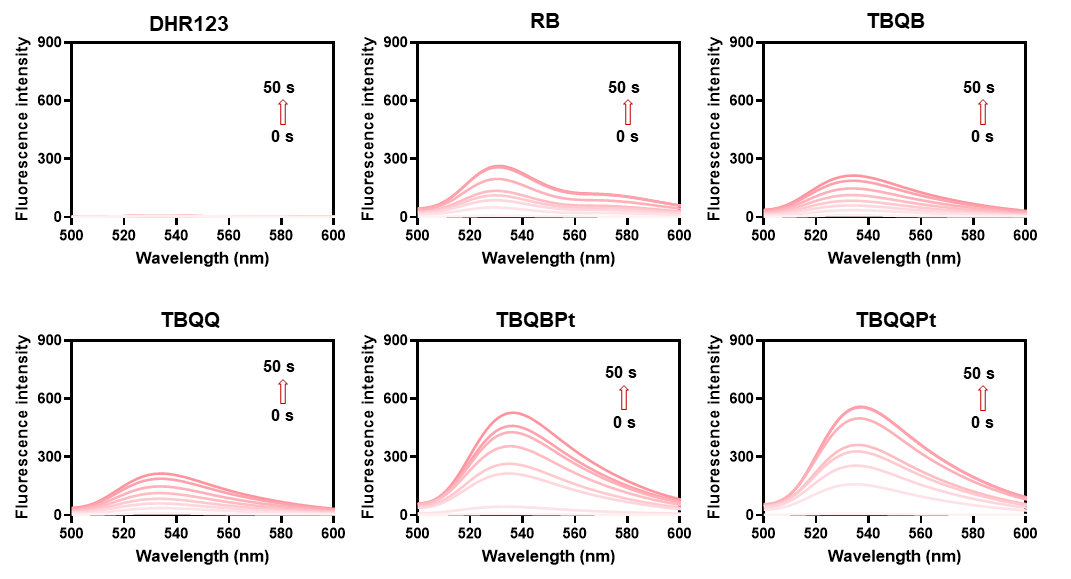


**Figure S19.** FL spectra of DHR123 in PBS in present of **TBQB, TBQQ, TBQBPt, TBQQPt**, **RB** and PBS under white light irradiation.


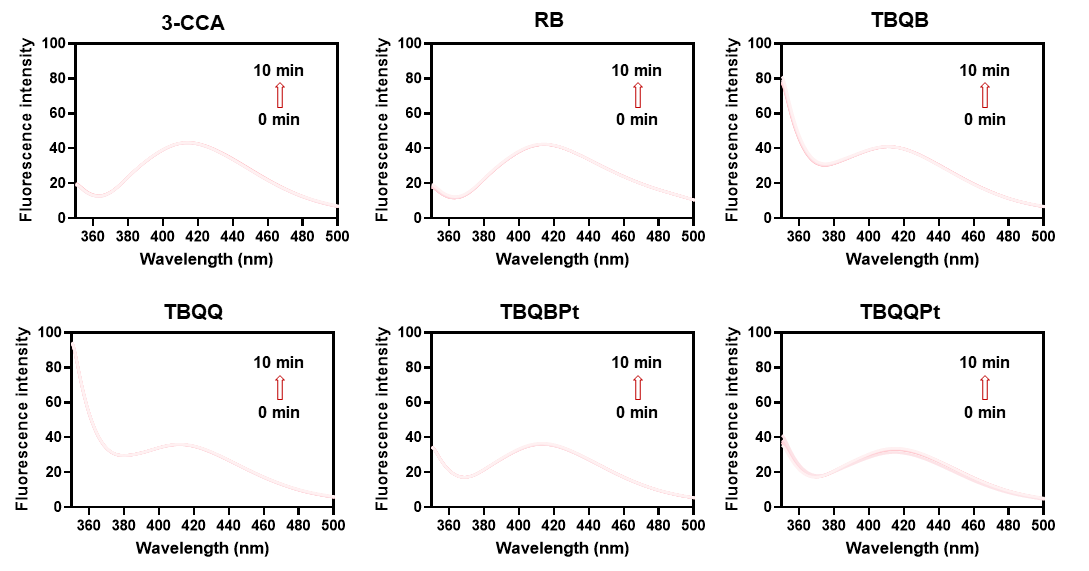


**Figure S20.** FL spectra of 3-CCA in PBS in present of **TBQB, TBQQ, TBQBPt, TBQQPt**, **RB** and PBS under white light irradiation.


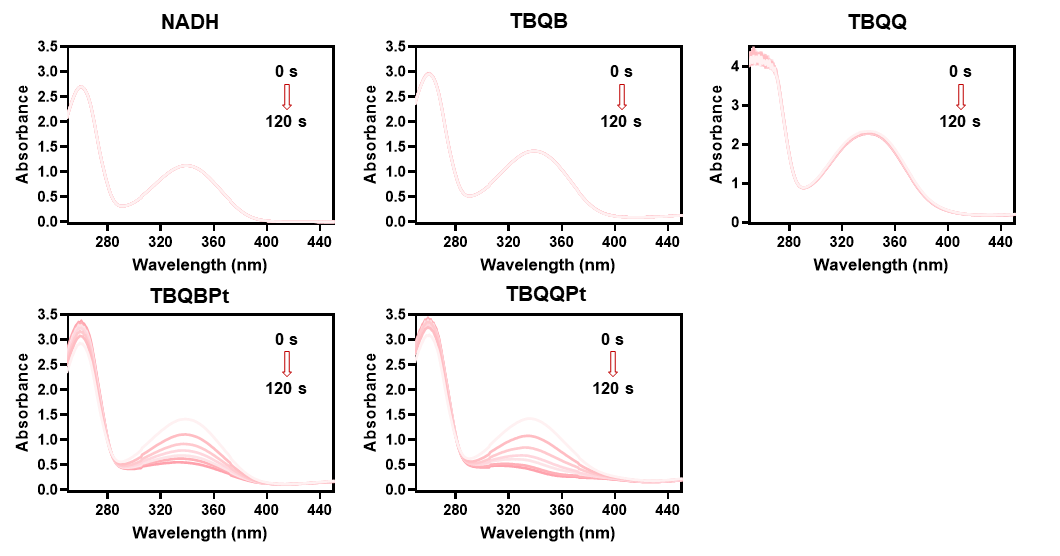


**Figure S21.** UV-vis spectra of NAD**+** in PBS in present of **TBQB, TBQQ, TBQBPt, TBQQPt**, **RB** and PBS under white light irradiation.


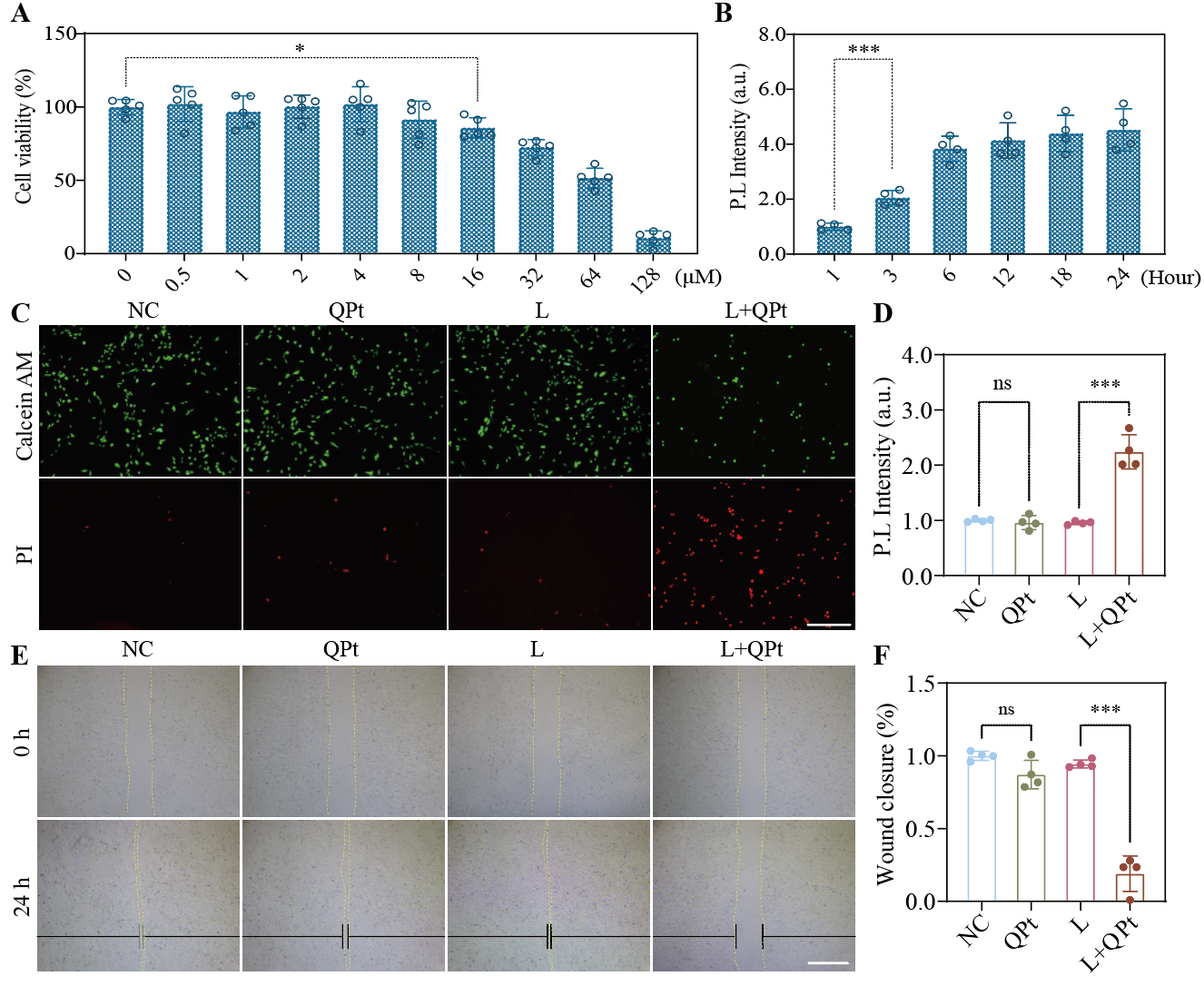


**Figure S22. A)** Cell viability was assessed in HKFs treated with increasing concentrations of QPt. **B)** Quantification of cellular uptake of QPt by confocal laser scanning microscopy. **C)** Calcein-AM/PI was used to detect cell viability across intervention groups. Scale bar: 100μm. **D)** Quantitative analysis of relative PI fluorescence intensity in cells, n = 4. **E)** Cell scratch assay was used to evaluate the cell migration ability across intervention groups. **F)** Quantification of wound closure rates at 24 hours post-intervention, n = 4. The results are expressed as mean ± SD. * *p* < 0.05, ** *p* <0.01, *** *p* < 0.001; ns, not significant.


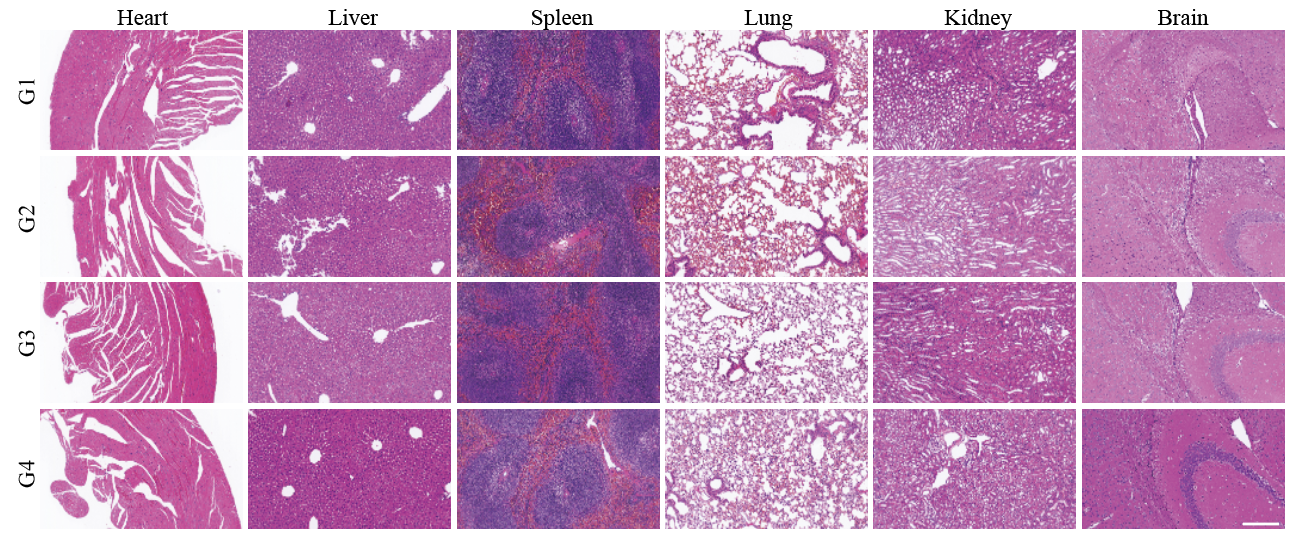


**Figure S23.** Pathological sections of major organs (heart, liver, spleen, lung, kidney and brain). Scale bar: 200μm.


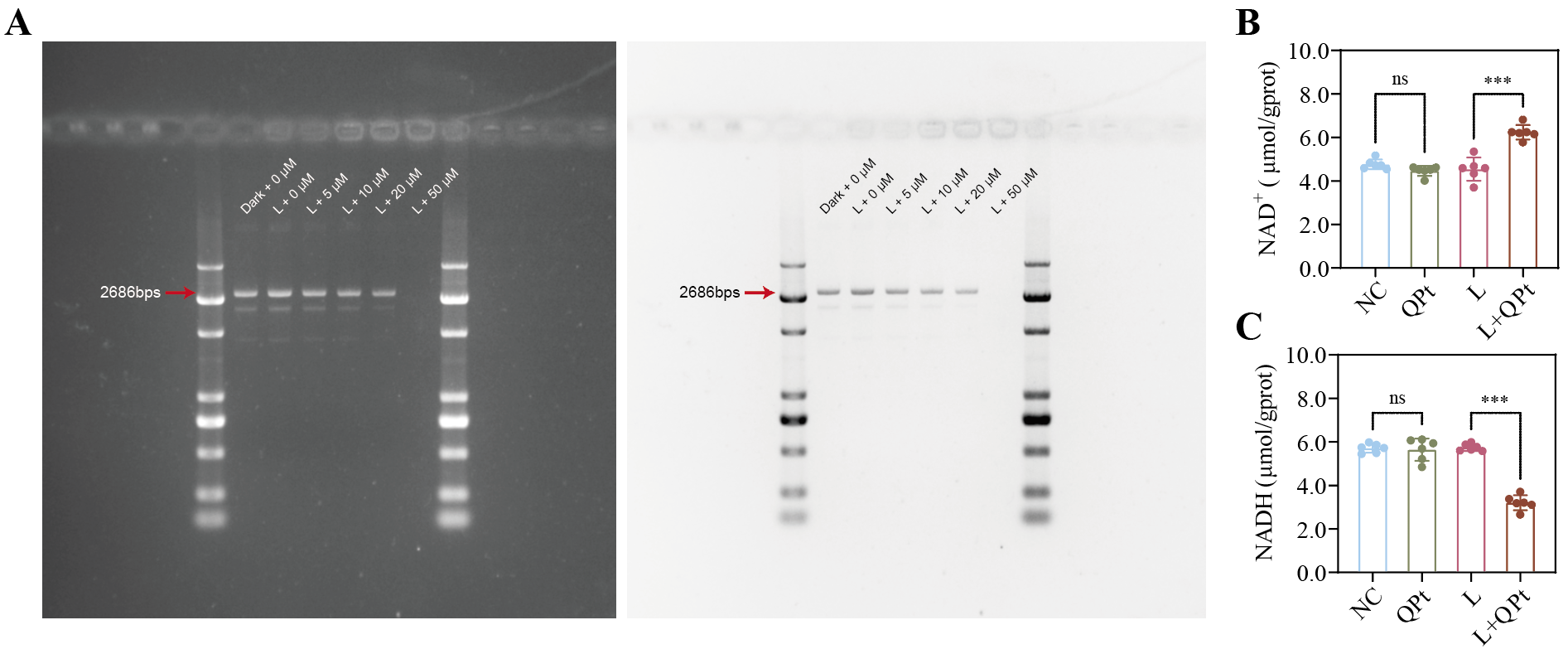


**Figure S24. A)** Agarose gel electrophoresis analysis of pUC-19 plasmid DNA (2686 bp) photocleavage induced by **TBQQPt** at various concentrations (0, 5, 10, 20, and 50 µM) under identical white light irradiation parameters (20 mW cm⁻², 5 min). **B-C)** Direct quantification of light-triggered intracellular nicotinamide adenine dinucleotide pool disruption in cellular. Intracellular absolute levels of NADH and NAD^+^ in HKFs after various treatments, n = 6. The results are expressed as mean ± SD. * *p* < 0.05, ** *p* <0.01, *** *p* < 0.001; ns, not significant.


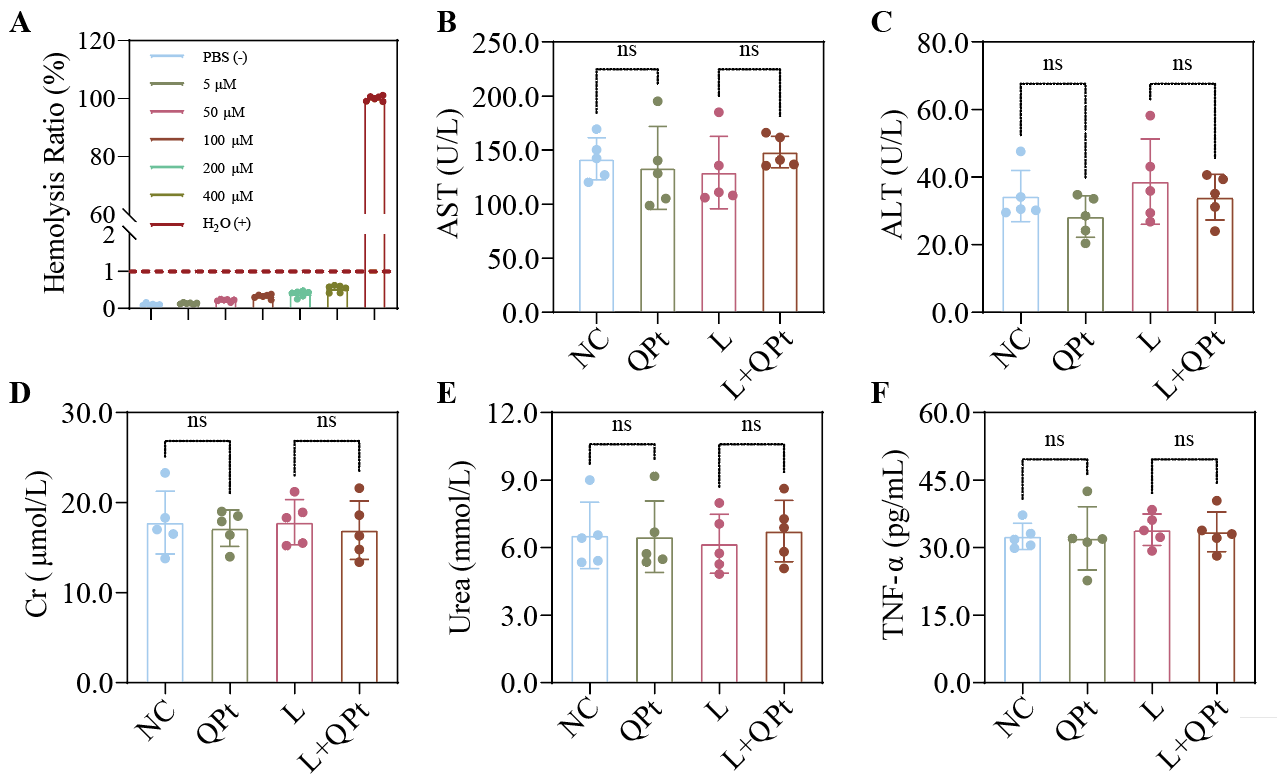


**Figure S25. A)** Quantitative in vitro hemolytic evaluation of **TBQQPt** against mice red blood cells at various concentrations (5, 50, 100, 200, and 400 µM) after 2 h of incubation. Deionized water and PBS served as positive and negative controls, respectively. Data are presented as mean ± SD (n = 6). **B-F)** In vivo systemic safety evaluation of **TBQQPt**. Serum biochemical parameters including hepatic functional markers ALT and AST, renal functional markers Urea and Cr, as well as the systemic inflammatory cytokine TNF-α, were measured, n = 5. The results are expressed as mean ± SD. * *p* < 0.05, ** *p* <0.01, *** *p* < 0.001; ns, not significant.


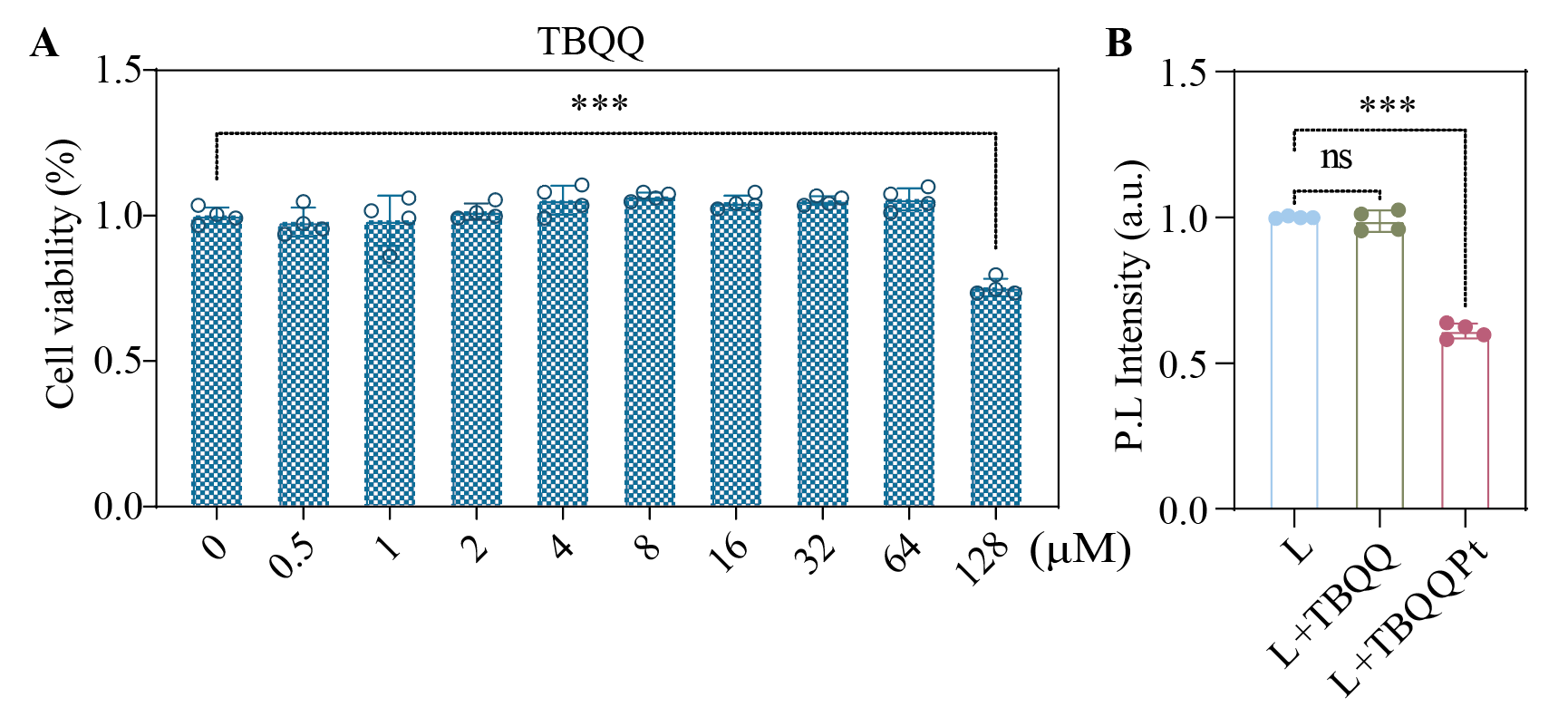


**Figure S26. A)** Dark cytotoxicity of the metal-free ligand **TBQQ** in HKFs after 24 h of incubation across a concentration gradient (0 to 128 µM). **B)** Cell viability of HKFs after running side-by-side phototherapeutic treatments with **TBQQ** (64 µM) or **TBQQPt** (8 µM) under identical white light irradiation (20 mW cm⁻², 5 min). Data are presented as mean ± SD (n = 4). The results are expressed as mean ± SD. * *p* < 0.05, ** *p* <0.01, *** *p* < 0.001; ns, not significant.


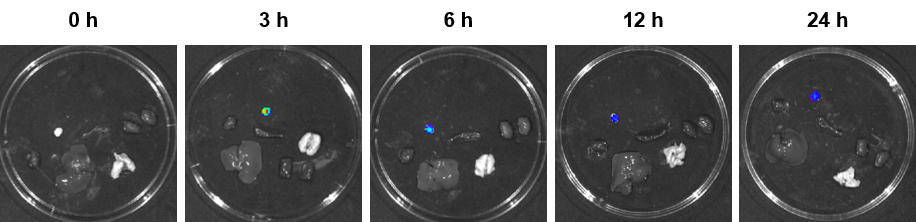


**Figure S27.** In vivo fluorescence imaging of **TBQQPt** following subcutaneous administration. Representative fluorescence images acquired at 0, 3, 6, 12, and 24 h after subcutaneous injection. The fluorescence signal remained localized at the injection site and gradually decreased over time, indicating prolonged local retention of **TBQQPt** in vivo.
